# Psychiatric and behavioural sequelae following encephalitis: a systematic review and meta-analysis

**DOI:** 10.1093/braincomms/fcag175

**Published:** 2026-06-18

**Authors:** Cameron J Watson, Jack B Fanshawe, Danish Hafeez, Katharine Lynch-Kelly, James Marsh, Yasmin Abdat, Hamish Hamilton, Dory A Ghanem, Wafaa Mowlabaccus, Brendan F Sargent, Hamilton Morrin, Ella Burchill, Cerian Jackson, Stephen McKeever, Belinda R Lennox, Adam E Handel, Benedict D Michael, Ava Easton, Matthew Butler, Thomas A Pollak

**Affiliations:** Social Genetic and Developmental Psychiatry Centre, Institute of Psychiatry, Psychology and Neuroscience, King’s College London, London SE5 8AF, UK; South London and Maudsley NHS Foundation Trust, London SE5 8AZ, UK; Department of Psychiatry, University of Oxford, Oxford OX3 7JX, UK; Oxford Health NHS Foundation Trust, Oxford OX3 7JX, UK; South London and Maudsley NHS Foundation Trust, London SE5 8AZ, UK; Department of Psychosis Studies, Institute of Psychiatry, Psychology and Neuroscience, King’s College London, London SE5 8AF, UK; South London and Maudsley NHS Foundation Trust, London SE5 8AZ, UK; Department of Psychosis Studies, Institute of Psychiatry, Psychology and Neuroscience, King’s College London, London SE5 8AF, UK; Royal Devon University Healthcare NHS Foundation Trust, Exeter EX2 5DW, UK; Department of Psychosis Studies, Institute of Psychiatry, Psychology and Neuroscience, King’s College London, London SE5 8AF, UK; South London and Maudsley NHS Foundation Trust, London SE5 8AZ, UK; Newcastle upon Tyne Hospitals NHS Foundation Trust, Newcastle upon Tyne, NE1 4LP, UK; Dykebar Hospital, NHS Greater Glasgow and Clyde, Paisley, PA2 7DE, UK; Department of Psychiatry, University of Oxford, Oxford OX3 7JX, UK; Oxford Health NHS Foundation Trust, Oxford OX3 7JX, UK; South London and Maudsley NHS Foundation Trust, London SE5 8AZ, UK; Department of Psychosis Studies, Institute of Psychiatry, Psychology and Neuroscience, King’s College London, London SE5 8AF, UK; Division of Psychiatry, University College London, London, W1T 7NF, UK; Liverpool Interdisciplinary Neuroscience Centre (LINC), University of Liverpool, Liverpool, L9 7AL, UK; Liverpool Interdisciplinary Neuroscience Centre (LINC), University of Liverpool, Liverpool, L9 7AL, UK; Department of Psychiatry, University of Oxford, Oxford OX3 7JX, UK; Oxford Health NHS Foundation Trust, Oxford OX3 7JX, UK; Oxford Health NHS Foundation Trust, Oxford OX3 7JX, UK; Nuffield Department of Clinical Neurosciences, University of Oxford, Oxford, OX3 9DU, UK; Liverpool Interdisciplinary Neuroscience Centre (LINC), University of Liverpool, Liverpool, L9 7AL, UK; Clinical Infection, Microbiology & Immunology, Institute of Infection, Veterinary and Ecological Sciences, University of Liverpool, Liverpool, CH64 7TE, UK; Encephalitis International, Malton, YO17 7DT, UK; Department of Neurology, Walton Centre of Neurosurgery and Neurology, Liverpool, L9 7LJ, UK; National Institute for Health and Care Research Health Protection Research Unit in Emerging and Zoonotic Infections, University of Liverpool, Liverpool, L69 7BE, UK; Clinical Infection, Microbiology & Immunology, Institute of Infection, Veterinary and Ecological Sciences, University of Liverpool, Liverpool, CH64 7TE, UK; Encephalitis International, Malton, YO17 7DT, UK; South London and Maudsley NHS Foundation Trust, London SE5 8AZ, UK; Department of Psychosis Studies, Institute of Psychiatry, Psychology and Neuroscience, King’s College London, London SE5 8AF, UK; South London and Maudsley NHS Foundation Trust, London SE5 8AZ, UK; Department of Psychosis Studies, Institute of Psychiatry, Psychology and Neuroscience, King’s College London, London SE5 8AF, UK

**Keywords:** encephalitis, outcome, meta-analysis, mental health, psychiatry

## Abstract

Survivors of encephalitis frequently experience chronic neuropsychiatric sequelae, yet the prevalence and patterns of mental health outcomes remain poorly characterized. We conducted a systematic review and meta-analysis to quantify the prevalence of psychiatric and behavioural symptoms following encephalitis. We also aimed to compare infectious with autoimmune encephalitis, explore specific aetiological associations as well as age-related differences. Following PRISMA guidelines, MEDLINE, EMBASE, PsycINFO, CINAHL, and PubMed were searched through 13 December 2024. Observational studies reporting psychiatric outcomes ≥3 months post-encephalitis were included. Two reviewers independently screened titles/abstracts and extracted data on symptom prevalence, study design, aetiology, and demographics. Random-effects meta-analyses estimated pooled prevalence of depression, anxiety, disinhibition, emotional instability, and other neuropsychiatric domains. Subgroup analyses compared infectious versus autoimmune causes and paediatric versus adult cohorts. Meta-regression assessed the influence of follow-up duration, sex percentage and cohort age. One hundred one studies (*n* = 4703 patients; weighted mean age 36.5 years) met inclusion. Across all aetiologies, pooled prevalence estimates included: depression, 26.9% (95% CI 22.2–32.3%); anxiety, 22.8% (95% CI 14.2–32.0%); disinhibition, 20.5% (95% CI 15.1–27.3%); emotional instability, 22.9% (95% CI 15.6–32.4%). Infectious encephalitis demonstrated higher rates of mood symptoms (75.2% versus 30.9% in autoimmune; *P* < 0.001). Meta-regression revealed that follow-up duration, mean cohort age, and female proportion influenced the prevalence of several neuropsychiatric symptoms. Heterogeneity was substantial across analyses, reflecting aetiologic, methodological, and demographic diversity in included studies. Psychiatric sequelae following encephalitis occur at rates comparable to neurological complications, with depression, anxiety, disinhibition, and emotional instability each affecting at least one-quarter of survivors. The substantial heterogeneity across studies highlights the need for prospective comparative cohorts, consistent diagnostic criteria, and the development of a standardized mental health outcome set to improve both care and research.

## Teaser

Through meta-analysis of encephalitis follow-up studies, Watson and Fanshawe *et al*. report that psychiatric symptoms occur at comparable frequency to neurological complications. Anxiety, depression, disinhibition, and emotional instability each affect at least one-quarter of survivors. There were no clear symptomatic distinctions in the neuropsychiatric trajectories of autoimmune and infectious aetiologies.

## Graphical Abstract

**Graphical Abstract:**
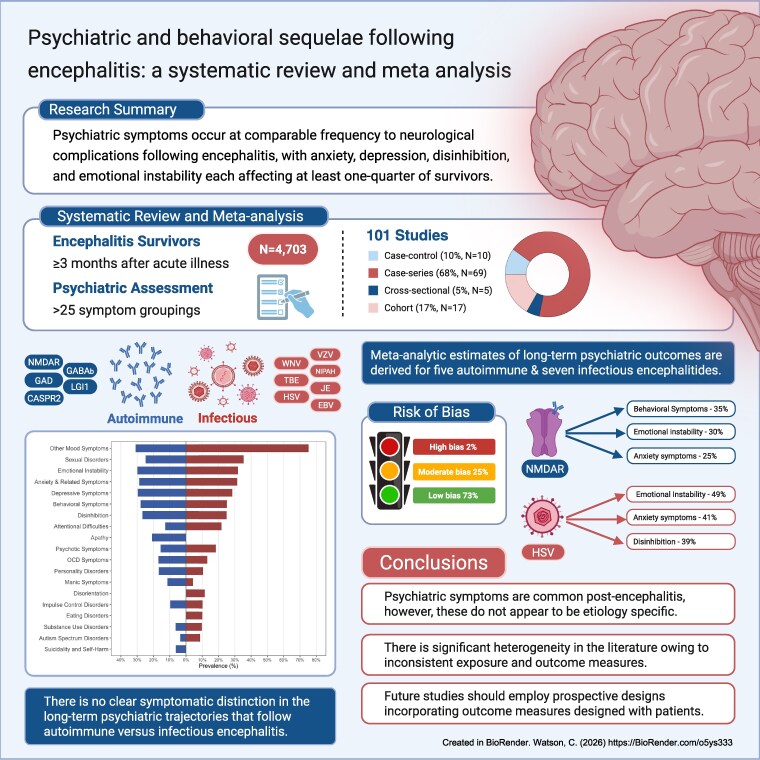

## Introduction

Encephalitis is defined as the presence of parenchymal inflammation of the brain with associated neurological dysfunction.^1^ Aetiologies of encephalitis are numerous, but commonly fall into two categories, infectious and immune-mediated, with the latter comprising post-infectious, paraneoplastic and autoimmune encephalitis.^1^ Advances in diagnosis and management have reduced encephalitis-related mortality, however, rates remain high, between 5% and 40%.^2-5^

Though survival has improved, substantial disability remains common, with major consequences for quality of life and socio-economic functioning^6-11^. Medical record data has also indicated a significantly increased risk of psychiatric disorders following encephalitis, including bipolar and psychotic disorders.^12^ Despite these findings, the characterization of long-term mental health outcomes following encephalitis has remained limited. Many existing studies have relied on coarse groupings, for example, aggregating all infectious or all autoimmune encephalitides. This potentially limits the ability to identify clinically meaningful differences between aetiologies and constrains efforts to develop more tailored approaches to diagnosis and management. Encephalitis-specific patient-reported outcome measurements (PROMs) are also lacking, resulting in uncertainty as to which symptoms most impact the lives of affected patients. The only disease-specific PROM in use is the LANTERN scale for LGI-1 encephalitis.^13^ Whilst the PROSE score is under development for use in autoimmune encephalitis more broadly,^14^ there is currently no disease-specific PROM available for infectious encephalitis.^8^ A deeper understanding of the psychiatric needs of this population could inform the development of standardized outcome measures, integrated healthcare services and targeted mental health support.

This systematic review and meta-analysis aimed to synthesize data on the frequency and nature of mental health outcomes following encephalitis. We sought to compare autoimmune and infectious causes at the group level and evaluate specific symptom prevalence across multiple encephalitis aetiologies. Characterizing the breadth and frequency of psychiatric and behavioural sequelae, we aimed to determine whether encephalitis subtypes are associated with specific psychiatric and behavioural outcomes, with the aim of improving understanding of long-term mental health impacts in affected individuals.

## Materials and methods

This systematic review is reported according to PRISMA guidelines (Supplementary Table 1).^15^ The study was not prospectively registered.

### Search strategy

In this systematic review and meta-analysis of symptom prevalence, authors searched OVID MEDLINE, PsycINFO and EMBASE, and CINAHL from database inception. The search was conducted on 13 December 2024, with the full search strategy presented in Supplementary Methods 1. Authors also examined reference lists of included articles and contacted significant researchers in the field to identify further works. Duplicate articles were identified automatically using Rayyan software,^16^ followed by manual deduplication comparing similar citations.

Peer-reviewed human studies of hospitalized patients with encephalitis of infectious, idiopathic or autoimmune aetiology was included (Table 1). Studies of patients with alternative or mixed pathology such as meningitis or meningoencephalitis were excluded. Studies reporting on patients with voltage-gated potassium channel (VGKC) encephalitis but not specifying the presence of Leucine-rich glioma-inactivated 1 (LGI-1) or Contactin-associated protein-like 2 (CASPR2) antibodies were excluded.^17^ NMDAR encephalitis studies reporting on psychiatric outcomes in the context of serum-only antibody confirmation without additional supportive investigations were also excluded. Outcomes were defined as mental health diagnoses or symptoms including clinician-rated, self-reported or assessed using validated questionnaires. Studies reporting generic psychiatric symptoms (e.g. psychiatric symptoms NOS) were excluded. Outcomes were included only if they were reported at least three months after hospitalization. If multiple follow-up points were reported, the latest was used for the analysis.

**Table 1:** Inclusion and exclusion criteria

Criterion	Inclusion	Exclusion
Population	Human patients of any age diagnosed with encephalitis (infectious, autoimmune, idiopathic)	Animal studies
Exposure	Confirmed any cause encephalitis by clinical and laboratory criteria (e.g. microbiology, PCR, autoantibody)	Patients with alternative or mixed pathology such as meningitis or meningoencephalitis. Studies reporting data from patients with VGKC encephalitis whereby antibodies to LGI-1 or CASPR2 were not reported. NMDA receptor encephalitis studies reporting on psychiatric outcomes in the context of serum-only antibody confirmation without additional supportive investigations.
Comparison	Either healthy controls or other neurological disease controls such as traumatic brain injury, stroke, or meningitis	Both controlled and uncontrolled studies were eligible, however control data was extracted where available.
Outcome	Any mental health outcome or diagnosis reported using clinician-rated, self-reported or assessed using validated questionnaires. Outcomes reported >/3 months post hospital discharge	Studies only reporting amalgamated symptoms such as ‘psychiatric symptoms’ without breakdown by domain
Study type	Peer-reviewed articles without date restriction reporting the results of observational and interventional studies with a total sample size of at least 5	Conference abstracts and other non-peer-reviewed data; Review articles; Case reports, case series with *n* < 5; Non-English language where translation not available

Two blinded reviewers independently evaluated article eligibility by sequentially reviewing titles and abstracts in parallel; where there was disagreement, a third reviewer arbitrated. Full texts were screened as per abstracts.

### Data extraction

Data were extracted by two blinded reviewers in parallel onto a pre-piloted spreadsheet. Where there were discrepancies between the data extracted, a third author arbitrated. Data were extracted on study design, study location, patient demographics, encephalitis aetiology, follow-up duration, symptom prevalence/rating, mental health diagnosis prevalence and the outcome measure used. Two trained reviewers assessed each paper using the Joanna Brigg’s institute tool for systematic reviews of specific study designs to assess risk of bias and applicability.^18^ In the case of disagreement, decisions were reconciled with a third reviewer. Authors were contacted for further details on data where required.

### Symptom groupings

Related symptoms were binned into broader domains to facilitate meta-analysis (e.g. delusions and hallucinations were grouped under ‘psychosis’). Full groupings in Supplementary Table 2.

### Statistical analysis

Meta-analyses were conducted using the *metafor* package in R (version 4.3.1).^19^ Due to anticipated heterogeneity, pooled prevalence estimates were calculated with a random-effects model on the logit scale (PLO measure). Prevalence estimates were pooled across all-cause encephalitis then stratified by autoimmune and infectious subgroups. Where at least two studies examined a specific symptom, further stratified analyses were conducted by specific aetiology [e.g. anti-N-methyl-D-aspartate receptor, (NMDAR)] and age group (adult versus paediatric). Studies were classified as paediatric if the median age was <18 years and the interquartile range or standard deviation did not cross this threshold. Differences in symptom prevalence between adults and children with all-cause encephalitis, and between patients of any age with either an infectious or autoimmune encephalitis were assessed using *Z*-tests of pooled estimates, with *P*-values derived. Where ≥ 5 studies were available, meta-regression was conducted to explore sources of heterogeneity, including cohort mean age, the proportion of female participants, and mean follow-up duration. In the NMDAR encephalitis group, we conducted a further sensitivity analysis to explore whether symptom prevalence estimates were influenced by the biofluid sampled to diagnose encephalitis. Groups were classified as either CSF-only confirmation or non-CSF-only. Heterogeneity was quantified using the *I*^2^ statistic.

## Results

### Study characteristics

**Figure 1:**
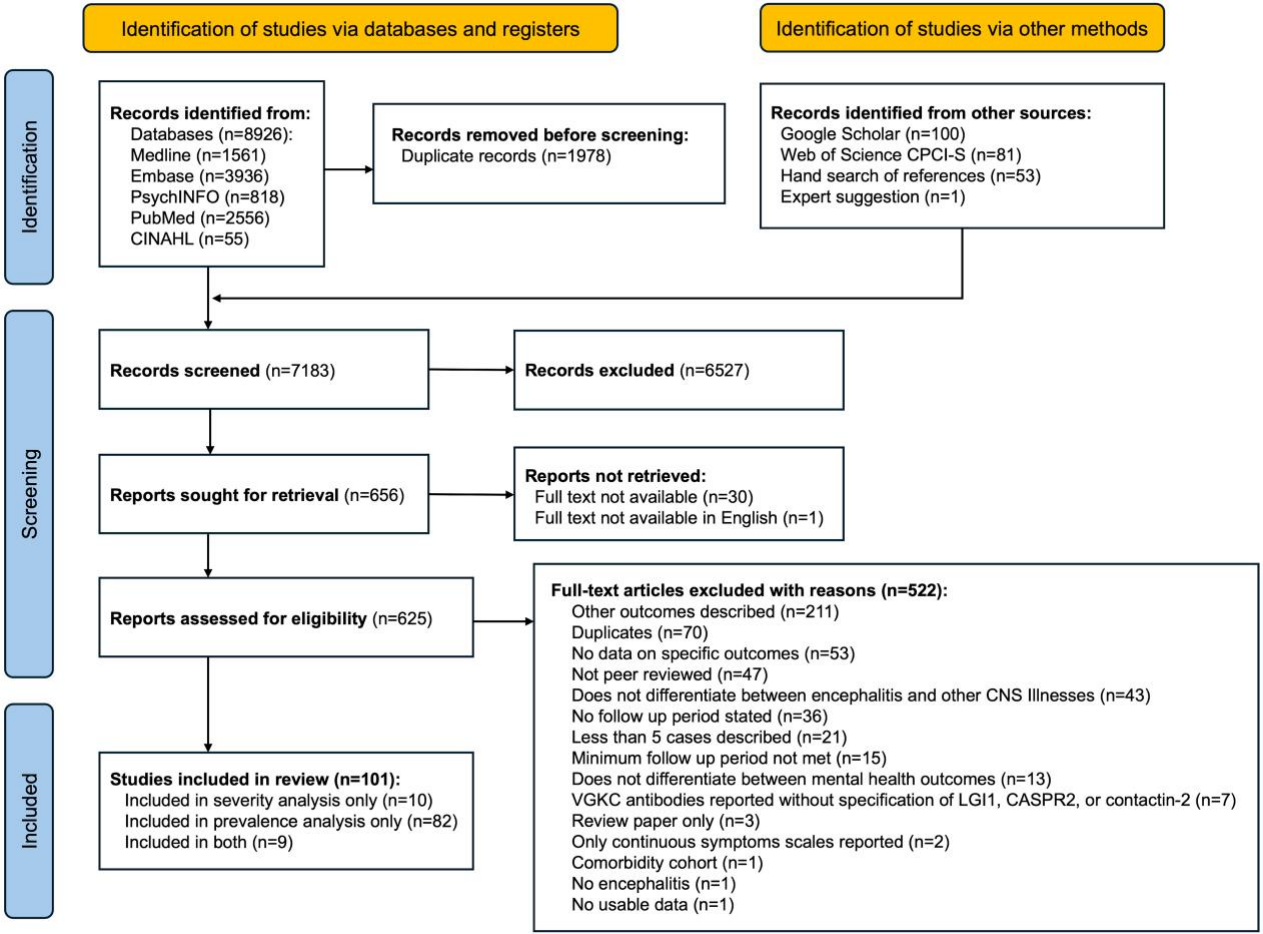
**PRISMA flow diagram.** The process of identification, screening, eligibility assessment and inclusion of studies in this systematic review and meta-analysis.

The search strategy yielded 9160 results. Screening and eligibility assessment resulted in 101 studies included of which 82 studies reported prevalence data only, nine reported both prevalence data and severity and 10 reported only severity data (Fig. 1).

Of the 101 studies, where data was available, a weighted mean age of 36.5 (SD 13.7) years was calculated across studies, comprising 4703 patients in total. Of these sex data were available for 4273, with 1841 males (39.1%) and 2432 females (51.7%) sex was not stated or derivable in 430 (9.1%). Characteristics of all included studies are presented in Supplementary Tables 3 and 4. In total, 38 studies reported on child participants only, 37 included adult participants only, and 24 reported mixed child and adult cohorts; data could not be derived for 2 studies.

**Figure 2:**
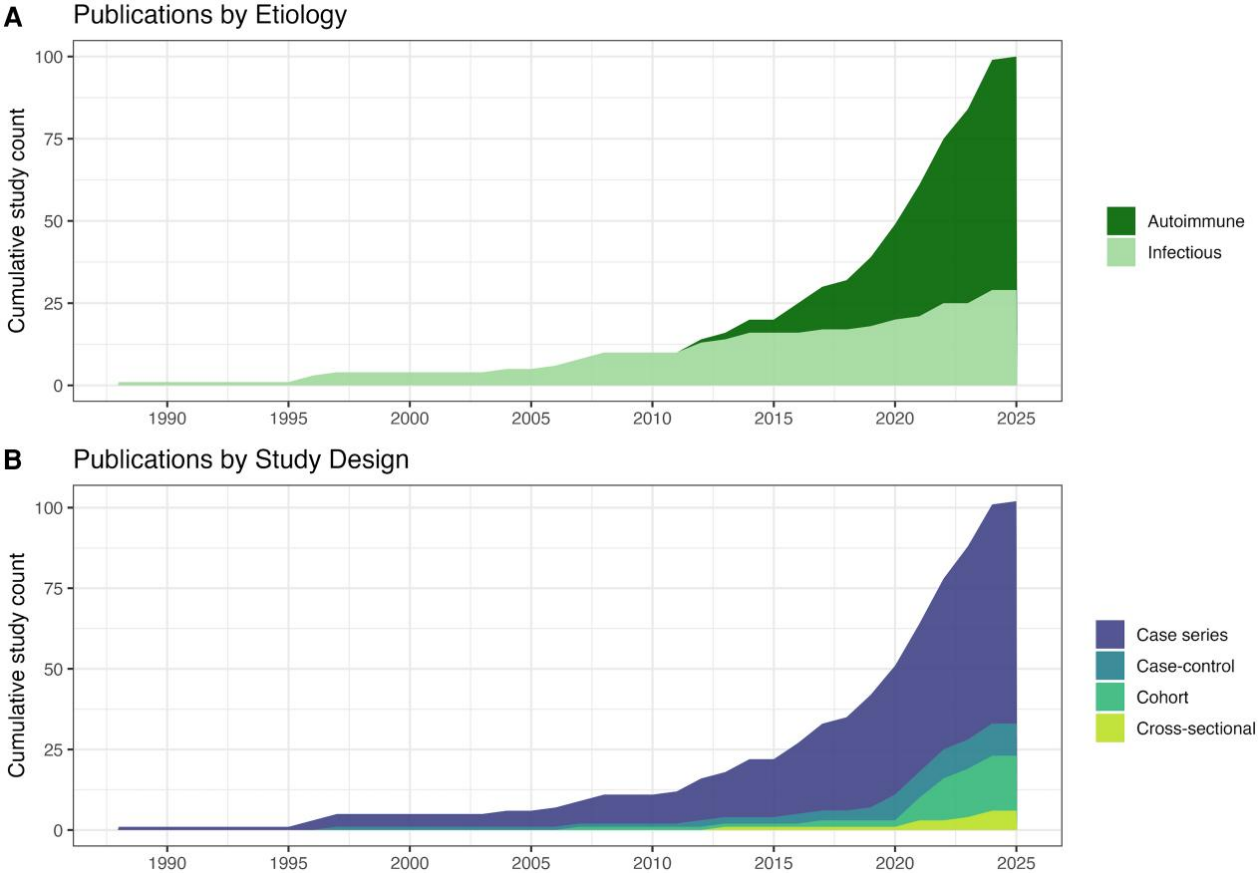
**Bibliometric patterns of publications on mental health outcomes post-encephalitis.** Cumulative number of publications reporting psychiatric or behavioural outcomes following encephalitis. Panel (**A**) shows cumulative publications stratified by encephalitis aetiology (autoimmune versus infectious). Panel (**B**) shows cumulative publications stratified by study design (case series, case-control, cohort and cross-sectional).

The most commonly studied symptom groups were depressive symptoms (53 studies), behavioural symptoms (39 studies), disinhibition (33 studies), anxiety and related symptoms (27 studies), and psychotic symptoms (25 cohorts). A total of 19 specific aetiological factors were reported, with anti-NMDAR (45 studies), Herpes Simplex Virus (HSV) (10 studies), anti-LGI1 (7 studies), and Japanese Encephalitis (5 studies) the most frequently examined (Fig. 2).

### All-cause encephalitis

Meta-analysis revealed that behavioural symptoms were the most prevalent psychiatric sequelae of all-cause encephalitis (Table 2), with an estimated prevalence of 27.7% across 43 cohorts and 1581 assessed patients. Depressive symptoms were also common, with 26.9% prevalence across 66 cohorts. Emotional instability was observed in 23.0% of participants, anxiety and related symptoms in 21.8%, disinhibition in 20.5%, and apathy in 19.4% of assessed cases.

**Table 2:** Meta-analytic estimate results for all cause encephalitis

Symptom	Prevalence (%)	95% Lower CI (%)	95% Higher CI (%)	Number of patients	I2	Number of cohorts
Depressive Symptoms	26.9	22.2	32.3	2649	82.3	66
Behavioural Symptoms	27.7	21.7	34.6	1581	82.4	43
Anxiety and Related Symptoms	21.8	14.2	32.0	1302	88.5	39
Disinhibition	20.5	15.1	27.3	1445	77.8	38
Psychotic Symptoms	11.0	7.7	15.6	1527	70.8	31
Emotional Instability	22.9	15.6	32.4	996	81.4	23
Attentional Difficulties	15.6	9.2	25.4	920	83.1	16
Other Mood Symptoms	21.5	7.7	47.5	632	95.7	9
OCD Symptoms	18.8	8.2	37.4	440	88.8	9
Apathy	19.4	8.3	39.1	139	70.0	8
Manic Symptoms	5.2	2.2	11.5	580	54.6	8
Eating Disorders	11.6	7.7	17.3	400	27.9	6
Suicidality and Self-Harm	6.5	2.5	16.0	183	34.9	6
Disorientation	11.6	6.6	19.6	259	43.7	6
Autism Spectrum Disorders	5.5	2.5	11.8	124	0.0	4
Substance Use Disorders	15.5	11.7	20.3	341	14.3	2
Personality Disorders	8.2	5.7	11.7	341	0.0	2
Impulse Control Disorders	14.7	2.9	49.8	10	0.0	2
Sexual Disorders	9.5	2.4	31.2	21	0.0	2

NOS, Not otherwise stated; OCD, obsessive compulsive disorder.

Subgroup analyses of adult-only and child-only cohorts allowed meta-analysis for 17 symptoms (Supplementary Table 5). Estimates for ASD (5.5%) were present only in children, whilst substance use (15.5%), impulse control disorders (14.7%), personality disorders (8.2%) and suicidality (3.7%) were meta-analysed only in adults. Estimates for 11 symptoms were available across both subgroups. The only symptom significantly different in prevalence between age groups was emotional instability at 26.3% in adults and 10.3% in children (*P* = 0.017), however this did not survive multiple testing correction (Supplementary Table 8).

### Infectious encephalitis

Meta-analytic estimates were generated for 17 neuropsychiatric symptoms following all-cause infectious encephalitis (Table 3). The most prevalent symptoms were ‘other mood symptoms’, with an estimated prevalence of 75.2%. This was followed by sexual disorders (35.4%), emotional instability (31.9%), and anxiety and related symptoms (31.4%), depressive symptoms were also common (28.4%). On meta-regression cohort age was associated with increased rates of anxiety (*β* = 0.08, *P* = 0.001) and psychotic symptoms (*β* = 0.02, *P* = 0.040); lower rates of disinhibition were associated with longer follow-up (*β* = −0.02, *P* = 0.042) (Supplementary Table 9). The proportion of females in a cohort was not significantly associated with the prevalence of any symptom.

**Table 3:** Meta-analytic estimate results for all cause infectious encephalitis

Symptom	Prevalence (%)	95% Lower CI (%)	95% Higher CI (%)	Number of patients	I2	Number of cohorts
Other Mood Symptoms	75.2	68.6	80.8	199	0.0	5
Sexual Disorders	35.4	29.2	42.1	220	0.0	7
Emotional Instability	31.9	19.4	47.6	761	86.3	11
Anxiety and Related Symptoms	31.4	13.7	56.9	354	88.2	14
Depressive Symptoms	28.4	20.7	37.7	1025	79.3	24
Disinhibition	24.9	15.4	37.7	889	83.8	18
Behavioural Symptoms	25.1	15.4	38.2	803	90.0	14
Attentional Difficulties	21.8	8.9	44.2	548	87.2	6
Psychotic Symptoms	18.3	6.6	41.8	790	92.5	8
OCD Symptoms	13.1	9.4	18.0	248	0	8
Disorientation	11.4	5.2	23.2	198	53.6	5
Personality Disorders	10.4	6.8	15.6	199	0.0	5
Impulse Control Disorders	10.2	6.6	15.3	199	0.0	5
Eating Disorders	9.9	3.3	26.3	50	18.4	3
Substance Use Disorders	9.8	4.5	20.2	199	20.4	5
Autism Spectrum Disorders	8.8	2.9	24.0	36	0.0	2
Manic symptoms	4.4	1.6	11.2	678	54.6	8

NOS, Not otherwise stated; OCD, obsessive compulsive disorder.

Subgroup analyses of adult and child only cohorts enabled meta-analysis for 15 symptoms (Supplementary Table 6). Estimates for attentional difficulties (32.8%), behavioural symptoms (25.1%) and autism spectrum disorders (ASD) (8.8%), were available only in children. Estimates of other mood symptoms (75.2%), anxiety (60.8%), sexual disorders (36.1%), emotional instability (35.9%), OCD symptoms (13.7%), personality disorders (10.4%), impulse control disorders (10.2%), substance misuse (9.8%) and manic symptoms (4.4%) were only available in adults.

### Autoimmune encephalitis

Group level meta-analytic estimates were generated for 17 neuropsychiatric symptoms (Table 4). The most prevalent was ‘other mood symptoms’, with an estimated prevalence of 30.9% across 11 cohorts. Emotional instability was also commonly reported (29.9%), followed by depressive symptoms in 29.6%. Anxiety and related symptoms were seen in 28.8%, while behavioural symptoms were estimated to be present in 27.8% of cases. Disinhibition was similarly common, with an estimated prevalence of 26.8%. Meta-regression revealed that rates of emotional instability were significantly greater in studies with longer follow-up (*β* = 0.09, *P* < 0.001), however, the reverse was true for anxiety (*β* = −0.04, *P* = 0.003), and depression (*β* = −0.04, *P* < 0.001). A higher proportion of females in the cohort was associated with lower rates of apathy (*β* = −0.05, *P* < 0.001). Increased cohort age was associated with increased disinhibition (*β* = 0.04, *P* = 0.013) and other mood symptoms (*β* = 0.06, *P* = 0.008) (Supplementary Table 10).

**Table 4:** Meta-analytic estimate results for all cause autoimmune encephalitis

Symptom	Prevalence (%)	95% Lower CI (%)	95% Higher CI (%)	Number of patients	I2	Number of cohorts
Other Mood Symptoms	30.9	11.4	61.0	349	90.6	11
Depressive Symptoms	29.6	23.3	36.7	1334	77.5	42
Anxiety and Related Symptoms	28.8	18.2	42.4	724	85.8	28
Emotional Instability	29.9	18.8	43.4	467	82.6	20
Behavioural Symptoms	27.8	20.0	37.3	684	76.5	27
Disinhibition	26.8	17.8	38.4	376	68.6	24
Sexual Disorders	24.9	15.4	37.5	58	0.0	4
Apathy	20.9	8.3	43.3	128	72.9	7
OCD Symptoms	17.0	6.9	36.1	108	59.8	8
Personality Disorders	16.8	8.9	29.3	58	0	4
Psychotic Symptoms	15.6	10.1	23.2	628	67.9	28
Attentional Difficulties	12.8	6.0	25.4	332	78.9	9
Manic Symptoms	11.5	6.9	18.6	137	0.0	7
Impulse Control Disorders	9.7	4.5	19.6	68	0.0	6
Substance Use Disorders	6.3	2.2	16.8	58	0.0	4
Suicidality and Self-Harm	6.3	1.8	19.9	154	45.3	5
Autism Spectrum Disorders	3.5	1.1	10.3	88	0.0	2

NOS, Not otherwise stated; OCD, obsessive compulsive disorder.

Subgroup analyses of adult-only and child-only cohorts enabled meta-analysis for 17 symptoms. Estimates for ASD (3.5%), were available only in children. Estimates for sexual disorders (24.9%), personality disorders (16.8%), manic symptoms (12.5%), impulse control disorders (9.7%), substance misuse (6.3%), and suicidality (3.7%) were available only in adults (Supplementary Table 7).

### Differences between infectious and autoimmune groups

**Figure 3:**
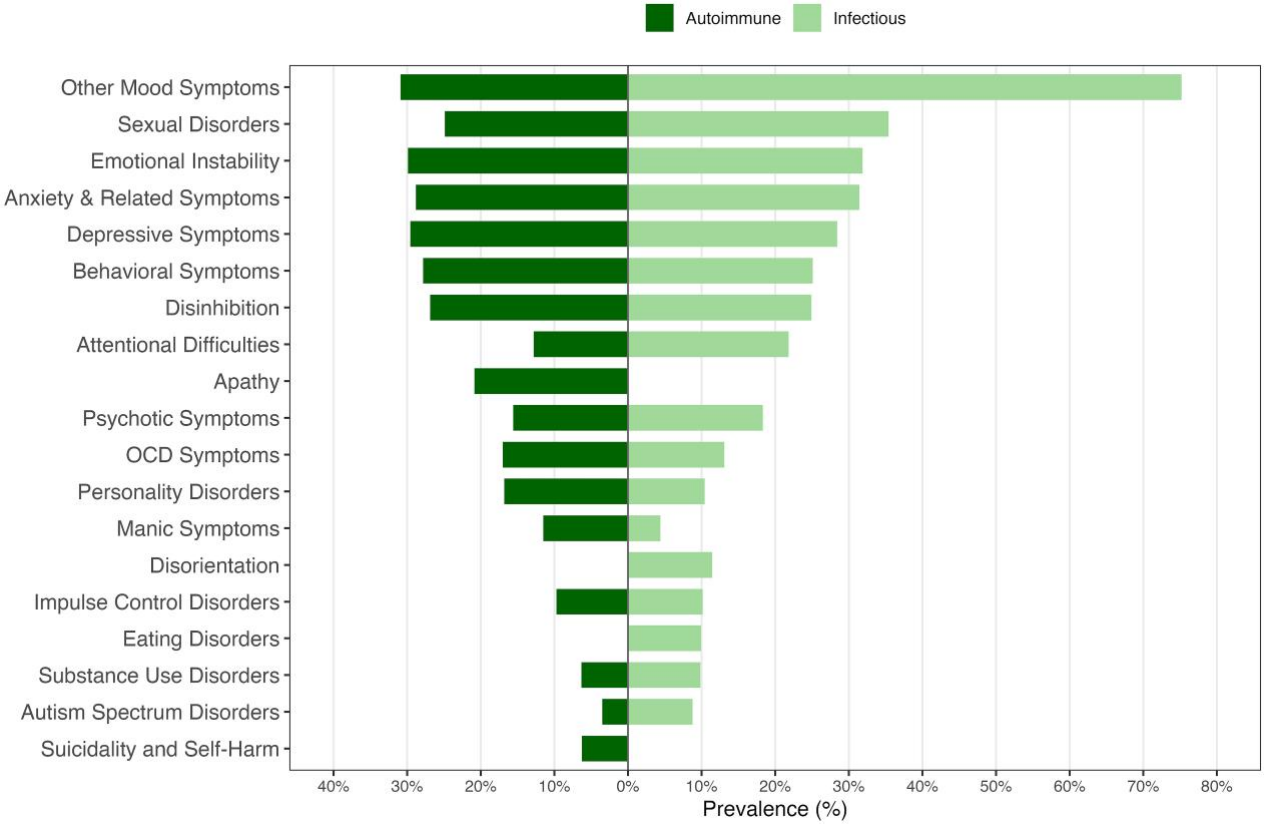
**Prevalence of symptoms post autoimmune and infectious encephalitis.** Pooled prevalence estimates of psychiatric symptoms following autoimmune and infectious encephalitis derived from meta-analysis of included studies. Symptom domains were eligible for meta-analysis if reported in two or more studies within a given aetiology group (autoimmune or infectious encephalitis). OCD, obsessive compulsive disorder.

Of 19 symptoms reported overall, apathy (20.9%), and suicidality (6.3%), was studied only in those with autoimmune encephalitis; disorientation (11.4%) and eating disorders (9.9%) were studied only in cases of infectious encephalitis. Of 15 symptom groupings meta-analysed across both infectious and autoimmune cases (Fig. 3), estimated prevalence rates were significantly different on *z*-score testing only for ‘other mood symptoms’ (75.2% in infectious versus 30.9% in autoimmune, *P* < 0.001), (Supplementary Table 11).

### Specific encephalitides—infectious aetiologies

**Figure 4:**
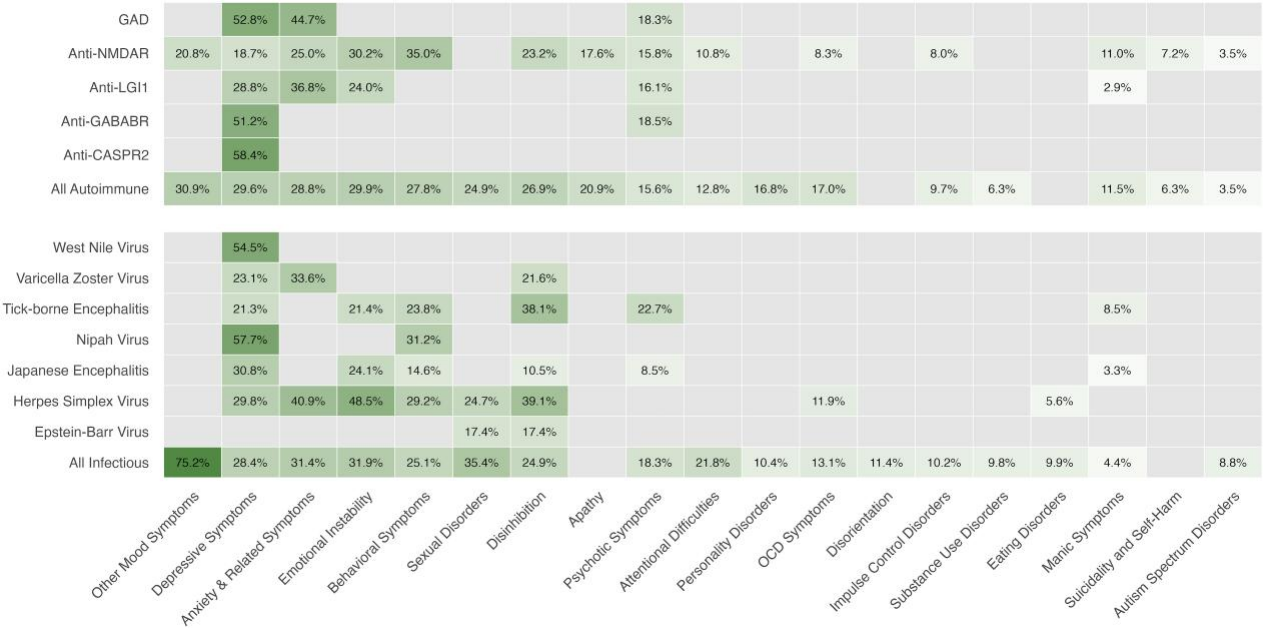
**Heat map of estimated symptoms prevalence post specific encephalitides.** Pooled meta-analytic estimates of psychiatric symptom prevalence across specific encephalitis aetiologies. The upper box contains autoimmune encephalitides while the lower summarizes infectious encephalitides. Symptom domains were eligible for meta-analysis if reported in ≥2 studies within a given aetiology. Cell colour represents the pooled prevalence estimate with darker shades of green reflecting greater prevalence. CASPR2, Contactin-associated protein-like 2; GABABR, Gamma-aminobutyric acid type B receptor; GAD, Glutamic acid decarboxylase; LGI-1, Leucine-rich glioma-inactivated 1; NMDAR, Anti-N-methyl-D-aspartate receptor.

Symptom prevalence estimates were available for seven infectious pathogens (Supplementary Table 12 and Fig. 4). Estimates were available for eight symptoms post HSV encephalitis, with emotional instability being the most prevalent (48.5%), followed by anxiety (40.9%) and disinhibition (39.1%). Estimates were available for six symptoms post Japanese encephalitis, with depressive symptoms the most prevalent (30.8%), followed by emotional instability (24.1%), and behavioural symptoms (14.6%). Estimates were available for six symptoms post tick-borne encephalitis, with disinhibition in 38.1% of cases, followed by behavioural symptoms (23.8%), and psychotic symptoms (22.7%). Results stratified by age group can be found in Supplementary Table 13.

In terms of symptom severity (Supplementary Table 4) Harris *et al.*,^20^ found that depression and anxiety mean scores were significantly higher in patients post HSV encephalitis than in healthy controls [13.81 (SD 8.13) versus 5.48 (SD 5.37) *P* < 0.001 and 10.04 (SD 9.51) versus 4.36 (SD 4.64) *P* = 0.004 respectively]. Fazekas *et al.*,^21^ reported a mean WHO-5 depression score of 13.4 (SD 5.8) post HSV encephalitis (Cut off 13 indicative of depression), Meyding-Lamadé *et al.*,^22^ reported mean HADS-D depressive score of 7.3 (SD 4.7) and a mean HADS-D anxiety score of 7.5 (SD 4.2) post HSV encephalitis (Score of >7 indicative of depression/anxiety respectively).

### Specific encephalitides—autoimmune aetiologies

Symptom prevalence estimates were available for five specific autoimmune aetiologies (Supplementary Table 14 and Fig. 4). Prevalence was estimated for 14 symptoms following NMDAR encephalitis, with behavioural symptoms the most common (35.0%), followed by emotional instability (30.2%) and anxiety symptoms (25.0%). Disinhibition (23.2%) and depressive symptoms (18.7%) were also commonly reported. Overall, 20 studies reported on symptoms in cohorts exclusively defined by CSF confirmation of NMDAR antibody status, 23 studies where biofluid sampled was either mixed or unknown, whilst only one study reported on a serum-only defined group. The latter paper reported on psychiatric outcomes in nine children with paediatric NMDAR encephalitis diagnosed using serum NR1 antibodies in a resource-constrained setting. These individuals displayed a markedly typical and severe phenotype including prominent hyperkinetic movement disorder requiring restraint as well as profound encephalopathy, with abnormal EEG found in all patients.^23^ The effect of fully CSF confirmed versus mixed or non-CSF confirmed cohorts influenced the prevalence of behavioural symptoms only, however, the association was nominally significant (Supplementary Table 15). Estimates were available for five symptoms post anti-LGI1 encephalitis; anxiety was estimated to have the highest prevalence (36.8%), followed by depressive symptoms (28.8%), and emotional instability (24.0%), with psychotic symptoms estimated to have been present in 16.1%. Estimates were available for three symptoms post anti-GAD encephalitis with depressive symptoms estimated to be the most prevalent (52.8%), followed by anxiety symptoms (44.7%), and psychotic symptoms (18.3%). Results stratified by age group can be found in Supplementary Table 16.

In studies reporting on symptom severity (Supplementary Table 4), Chen *et al.*,^24^ reported no significant difference between healthy controls and patients post NMDAR encephalitis in self-rating depression or anxiety scales (*P* = 0.425 and *P* = 0.133 respectively). Guasp *et al.*,^25^ reported significantly higher median HDRS and YMRS scores in patients compared with controls [6 (IQR 5–10) versus 0 (IQR 0–1), *P* < 0.001, and 4 (IQR 2–7) versus 0 (0-0), *P* < 0.020 respectively]. No significant difference was evident on median PANSS positive or negative subscales but was present for PANSS general and total score. Wang *et al.*,^26^ reported no significant difference in self-rating depression or anxiety scales in patients and healthy controls (*P* = 0.456 and *P* = 0.493 respectively). McKeon *et al.*,^27^ reported significantly increased mean anxiety scores in patients compared with healthy controls but no difference was found on depression scores (*P* = 0.048 and *P* = 0.303 respectively). Cai *et al.*,^28^ reported significantly higher anxiety and depression scores in patients versus healthy controls (*P* = 0.004 and *P* = 0.010 respectively). Ariño *et al.*,^29^ reported significantly increased mean depressive, manic, and psychotic symptoms, in patients compared with healthy controls (*P* < 0.001 for all).

Galioto *et al.*,^30^ reported no significantly higher symptom scores for anxiety or depression in patients post LGI1 encephalitis compared with healthy controls, amnestic mild cognitive impairment or temporal lobe epilepsy (*P* = 0.120 and *P* = 0.820 respectively). Sola-Valls *et al.*,^31^ reported significantly higher median combined anxiety or depressive symptoms (HADS) in patients compared with healthy controls [5.5 (IQR 3–9) versus 2 (IQR 0–5) *P* = 0.010]. Results from studies without control groups, as well as those reporting mixed encephalitis cohorts versus controls, are presented in Supplementary Table 4.

### Risk of bias

Overall, 74 studies (73.2%) were assessed as having low risk of bias, 25 studies (24.8%) as moderate risk of bias, and 2 studies (2.0%) as high-risk of bias (Supplementary Figs 1–3). Themes which were found to have a consistently low risk of bias across all study types included appropriate use of statistical analyses, specificity and clarity of inclusion criteria in case series. There was a significant variety of methods and criteria used to measure neuropsychiatric outcomes amongst case series, increasing the risk of bias and making the comparison of rates of symptoms or outcomes challenging across studies, as the diagnostic criteria and thresholds required to define neuropsychiatric outcomes frequently differed between studies.

The standardization, and validity of the definition of encephalitis diagnosis across studies also varied, with not all studies using standardized clinical testing for all participants, such as CSF analysis, or internationally validated criteria, such as the Graus criteria,^32^ when defining cases. While demographic and clinical information was assessed as being reported to a sufficient level across the majority of studies, a lack of detailed demographic information both at baseline, and at the time of outcome, was a recurrent theme. Information on participants’ geographical location, socio-economic status, level of education, employment, living arrangements, drug, nicotine and alcohol use was lacking from almost all studies, meaning this was frequently not matched or accounted for when assessing and reporting neuropsychiatric outcomes.

## Discussion

This review presents, to our knowledge, the largest and most comprehensive analysis of psychiatric and behavioural outcomes following encephalitis. We found a broad spectrum of sequelae, with depressive symptoms and behavioural disturbances the most common, each affecting around 27% of survivors. Anxiety, disinhibition, and emotional instability were also frequent, affecting roughly one in five patients.

### Infectious versus autoimmune aetiologies

Most existing studies have focused on individual aetiologies, commonly anti-NMDAR or HSV encephalitis, limiting opportunities for comparison across subtypes. In contrast, our synthesis aggregated data across a wide range of autoimmune and infectious aetiologies, allowing pooling of estimates across a range of outcomes. We found broadly similar profiles of psychiatric sequelae across infectious and autoimmune aetiologies, with most prevalence estimates not significantly differing between the two subgroups.

Anxiety and related symptoms were nearly identical across the groups (31% infectious versus 29% autoimmune), while depressive symptoms occurred at a comparable frequency (28% versus 30%). This similarity aligns with a recent large international survey, reporting no significant differences in rates of psychiatric symptoms between infectious and autoimmune encephalitis survivors.^33^ In autoimmune encephalitis, our estimated depressive symptom prevalence (30%) is lower than the 57% reported in a recent, well powered, prospective mixed-autoimmune cohort.^20^ Such discrepancy likely reflects methodological differences in our included studies, including the timing of assessments, symptom assessment tools, and cohort composition.

Relatively few studies have examined psychiatric outcomes in mixed infectious cohorts, however, in one Finnish study at a mean 81 months post-discharge 8/12 (67%) individuals had neuropsychiatric sequelae: depression (*n* = 4), aggression (*n* = 2), anxiety and bipolar disorder (both *n* = 1).^34^ Overall, our prevalence estimates for neuropsychiatric outcomes across both infectious and autoimmune causes have a comparable residual effect to more well-documented neurological and functional sequelae. A recent review found that 29% had a poor outcome in NMDAR encephalitis at one year (modified Rankin Scale >2).^35^

Despite overall similarities, our analysis did reveal some notable differences between infectious and autoimmune encephalitis. Most prominently, other mood symptoms, a category that included ‘non-specific’ mood symptoms like ‘Mood problem’ or ‘Other mood symptoms’, were reported in 75% of patients following infectious encephalitis, compared with 31% in autoimmune cases. Several symptom categories were also exclusive to only one subgroup.

Apathy and suicidality were reported solely in autoimmune encephalitis cohorts, whilst disorientation was described at follow-up in infectious cases only. These differences could reflect neurobiological differences across subtypes but might also represent reporting bias. There has been a notable increase in interest around neuropsychiatric aspects of autoimmune conditions likely resulting in more thorough examination of behavioural outcomes in these cohorts. Suicidality specifically is poorly explored across many medical settings, which resonates further with survey findings that 38% of encephalitis survivors had contemplated suicide while 4% had attempted it after their illness.^33^ Moreover, a 2020 study in Denmark revealed that suicide rates in patients diagnosed with encephalitis was nearly double that of people without a neurological disorder (39.7 per 100 000 person years).^36^

### Specific encephalitides show varied clinical profiles

#### Herpes simplex virus

Herpes simplex virus (HSV) encephalitis survivors demonstrated high rates of emotional instability (49%), anxiety (41%) and disinhibition (39%) in our study. Pronounced personality changes, irritability, mood swings, and impulse control problems are consistent with the tendency for HSV encephalitis to preferentially affect the medial temporal and orbitofrontal regions.^21^ Psychiatric symptoms in HSV are also often comorbid with a range of cognitive deficits and as such require considered aetiological exploration.^20^ Notwithstanding this, studies have reported that anxiety and depression often persist in HSV, even once deficits on neuropsychometric testing have normalized.^20^

#### Japanese encephalitis

Japanese encephalitis (JE) virus is a leading cause of encephalitis in Asia which can result in diffuse brain injury with a predilection for the basal ganglia and thalami.^37^ Our analysis provided estimates for six neuropsychiatric outcomes after JE. The most prevalent were depressive symptoms (31%), emotional instability (24%) and behavioural problems (15%). These rates, while significant, were somewhat lower than those seen following HSV. Given that JE is often associated with severe neurological deficits, it is possible that neuropsychiatric manifestations are relatively underreported.

#### Tick-borne encephalitis

Tick-borne encephalitis (TBE) was associated with a high prevalence of disinhibition (38%), as well as behavioural (24%) and psychotic symptoms (23%). The prevalence of psychosis in TBE was actually the highest amongst all examined aetiologies, though was only assessed in two cohorts. Nonetheless, these findings are supported by studies reporting on a post-TBE-encephalitic syndrome ranging from 10 to 58% in survivors, characterized by difficulty with concentration, memory and emotional regulation.^6,38,39^ Psychiatric and cognitive problems are frequent in these individuals with one study highlighting that 44% of those afflicted required psychiatric treatment after discharge.^40^

#### Anti-NMDA receptor encephalitis

Anti-NMDAR encephalitis can manifest in a breadth of acute psychiatric symptoms, with many cases seeing a psychiatrist as their first clinical contact.^41^ Our meta-analysis of 17 cohorts found the most prevalent sequelae were behavioural symptoms (35%), emotional instability (30%), anxiety (25%), and disinhibition (23%), with depressive symptoms also seen in nearly 19% of cases. This is consistent with prior longitudinal work. For example, Guasp *et al*.^25^ followed 28 patients for 1 year and reported psychiatric or behavioural impairments in 86% at 4 months and 44% at 12 months after symptom onset. Heine *et al.*^42^ has similarly reported that around 44% of patients reported depression and 60% anxiety at a median of 2.3 years post-onset.

Given the relatively young age of onset and improving treatment options, Anti-NMDAR encephalitis can be associated with good neurologic recovery.^35^ Our data reinforces, however, that neurologic ‘recovery’ does not equate to the absence of other potentially burdensome symptoms. In fact, one study using patient-reported outcomes found that 4 years after their acute illness, NMDAR encephalitis survivors had significantly lower quality-of-life scores, particularly in psychosocial domains compared with population norms.^43^

#### Anti-LGI1 encephalitis

Encephalitis associated with LGI1 antibodies typically affects older adults, who often present with limbic features and faciobrachial dystonic seizures.^44,45^ Various psychiatric and behavioural symptoms have been reported as part of the presentation in the acute phase, including psychosis, depression and hypomania.^46,47^ In one prospective cohort study, however, the only psychiatric symptoms remaining at a median 15 months post immunotherapy were mild depressive symptoms in 45%.^48^ Notably these patients also had concurrent cognitive impairment, compared with only 36% of those without depression. Though comparatively fewer symptom estimates could be calculated for LGI1 compared with other aetiologies, our meta-analytic estimates suggest that anxiety (37%), depressive symptoms (29%) and emotional instability (24%) may still be prominent following acute encephalitis.

### Age-related differences and meta-regressions

Our meta-regressions of infectious and autoimmune encephalitis showed that older age predicted higher rates of mood symptoms and anxiety, whilst ADHD and ASD were only reported in child cohorts. The findings may reflect differing aetiological spectra and the effect of encephalitis on neurodevelopment in childhood cohorts.^49^ Older survivors may have less neuroplasticity or face more cumulative stressors (e.g. loss of function, chronic pain, and menopause) that predispose them to mood disorders. This age effect persisted across aetiologic subgroups, indicating it is not simply due to disease type.

### Limitations

This synthesis reflects significant heterogeneity in the underlying literature, particularly in how psychiatric diagnoses and symptoms were ascertained. Some studies relied on structured interviews or validated diagnostic criteria, while others used unstandardized clinician codes, subjective notes, or caregiver/self-reports. Although self-reported symptoms offer insight into lived experience, they lack diagnostic specificity and are prone to misclassification. In many cases, it was unclear whether reported symptoms met formal thresholds for psychiatric disorders, limiting the interpretability and comparability of findings across studies and aetiologies. Alongside heterogeneity in outcome capture, our risk of bias analyses also revealed high variability in the use of standardized, validated criteria for encephalitis diagnosis.

Moreover, few studies employed protocolized, prospective designs. Follow-up durations varied, and outcome measures were inconsistent or poorly defined. Only a minority included healthy or neurological controls, restricting our ability to draw causal inferences. The recent development of the PROSE tool (Patient-Reported Outcome Scale for Encephalitis) is a promising step towards more standardized and meaningful symptom measurement, particularly in capturing neuropsychiatric outcomes beyond acute hospitalization.^14^

These inconsistencies in the source literature translated into marked statistical heterogeneity in our meta-analyses, even after stratification by aetiology or age group. Methodological variation, including differing case definitions, diagnostic practices, and follow-up intervals, limited our ability to pool data consistently. Clinical heterogeneity, such as differences in encephalitis aetiology, variation in baseline disease severity, treatment protocols (e.g. immunotherapy, antivirals), and healthcare settings, may also have contributed. We employed subgroup and sensitivity analyses to mitigate heterogeneity where possible; however, residual heterogeneity remained substantial for many estimates, possibly a result of the subsequently reduced sample sizes in these analyses. As discussed above the use of standardized case definitions and outcome measures will allow for future studies to enhance interpretability and generalizability of findings.

### Clinical implications and future directions

This synthesis confirms that psychiatric symptoms are common following many forms of encephalitis. Given the breadth and presence of symptoms at a range of follow-up points, we recommend routine mental health screening at structured intervals (e.g. 3, months, 6 months, 12 months, and to consider annual screening thereafter). In a multi-centre case-control study of HSV, other infectious, and autoimmune encephalitis survivors assessed at >1-year post-discharge, patients reported significantly higher HADS-Depression and HADS-Anxiety scores than healthy controls, despite normalization of neuropsychological test performance in intelligence and executive function.^20^ This emphasizes the importance of screening for psychiatric symptoms even in the absence of other impairments.

Validated tools such as the PHQ-9 and GAD-7 may facilitate early identification of psychiatric sequelae, however, current evidence is limited by the lack of protocol informed, prospective multicentre cohort studies analyzing neuropsychiatric outcomes. The SAPIENCE study, focusing on NMDAR encephalitis, incorporating patient and carer informed PROMS and detailed neuropsychological assessment,^50^ will partially fill this gap and provide an invaluable resource on prevalence and trajectory of neuropsychiatric symptoms. However, further studies of this type of multiple encephalitis aetiologies are required to compare outcomes between aetiologies and allow for informed clinical pathways to be developed.

Given the broad domains of mental health difficulties seen following encephalitis, our findings support the integration of neuropsychiatric expertise into long-term care pathways for survivors, as well as the perspectives of people with lived experience of post-encephalitis mental health problems in the design of research in this area. Few studies have examined predictors of psychiatric outcomes following encephalitis, and as such the patients to prioritize for referral remains unclear. Given the complex nature of primary psychiatric conditions, future studies should examine not only social determinants of poor outcomes, but also associations between neuroimaging and inflammatory profiles. Identifying these factors may enable early stratification of clinically high-risk cohorts and targeted intervention.

Interventional studies, both pharmacological and psychotherapeutic, are strikingly absent, despite such substantial psychiatric morbidity. Moreover, evidence on the impact of neurorehabilitation on psychiatric outcomes is limited. Understanding how improvement in one domain (e.g. cognition) may influence others (e.g. mood or behaviour) is essential to designing integrated treatment strategies, optimizing recovery and holistic care. Preliminary evidence suggests that ongoing neuropsychiatric involvement may improve outcomes in some subgroups (e.g. increased odds of return to work or school after NMDAR antibody encephalitis),^43^ but further studies are needed to confirm this and to guide best practices for long-term follow-up. Given the importance of mental health and wellbeing to all aspects of quality of life, it is striking that within the encephalitis literature there remains a dearth of research into the effect of encephalitis on important psychological and social factors, including parenting, caring, family dynamics, employment, social networks, intimacy and sexuality.

## Conclusion

Neuropsychiatric sequelae of encephalitis are common, especially depression, anxiety, behavioural disturbances, and emotional instability, affecting at least one-quarter of survivors. Proactive recognition of these sequelae is crucial for appropriate treatment to be instigated and avoid impacts on the patient's quality of life. Further prospective cohort studies utilizing comprehensive validated outcome sets are required to elucidate prognostic factors for neuropsychiatric symptoms, how these symptoms differ between encephalitis aetiologies and to ultimately inform clinical pathways and future interventional studies.

## Supplementary Material

fcag175_Supplementary_Data

## Data Availability

Data used in the current study are available from the corresponding author upon reasonable request. Full analytic code is available at: https://github.com/CameronWatson2020/encephalitis_psych_meta.
