# Supplementary material for: Psychiatric and behavioural sequelae following encephalitis: a systematic review and meta-analysis
Source: Brain Commun. 2026 Jun 18;8(3):fcag175. doi: 10.1093/braincomms/fcag175 (PMC13276490; doi:10.1093/braincomms/fcag175)
Supplement: fcag175_Supplementary_Data [file fcag175_supplementary_data.pdf]

Supplementary materials for psychiatric and behavioral sequelae following encephalitis: a systematic review and meta-analysis

Section and Topic	Item #	Checklist item	Location where item is reported
**TITLE**			
Title	1	Identify the report as a systematic review.	Title page
**ABSTRACT**			
Abstract	2	See the PRISMA 2020 for Abstracts checklist.	Abstract
**INTRODUCTION**			
Rationale	3	Describe the rationale for the review in the context of existing knowledge.	Introduction (page 4)
Objectives	4	Provide an explicit statement of the objective(s) or question(s) the review addresses.	Introduction (page 4)
**METHODS**			
Eligibility criteria	5	Specify the inclusion and exclusion criteria for the review and how studies were grouped for the syntheses.	Methods (pages 5 & 6)
Information sources	6	Specify all databases, registers, websites, organisations, reference lists and other sources searched or consulted to identify studies. Specify the date when each source was last searched or consulted.	Methods (page 5)
Search strategy	7	Present the full search strategies for all databases, registers and websites, including any filters and limits used.	Supplementary Methods 1
Selection process	8	Specify the methods used to decide whether a study met the inclusion criteria of the review, including how many reviewers screened each record and each report retrieved, whether they worked independently, and if applicable, details of automation tools used in the process.	Methods (page 6)
Data collection process	9	Specify the methods used to collect data from reports, including how many reviewers collected data from each report, whether they worked independently, any processes for obtaining or confirming data from study investigators, and if applicable, details of automation tools used in the process.	Methods (pages 5 & 6)
Data items	10a	List and define all outcomes for which data were sought. Specify whether all results that were compatible with each outcome domain in each study were sought (e.g. for all measures, time points, analyses), and if not, the methods used to decide which results to collect.	Methods (page 5 & 6); Supplementary Table 2
	10b	List and define all other variables for which data were sought (e.g. participant and intervention characteristics, funding sources). Describe any assumptions made about any missing or unclear information.	Methods (page 5 & 6)

Study risk of bias assessment	11	Specify the methods used to assess risk of bias in the included studies, including details of the tool(s) used, how many reviewers assessed each study and whether they worked independently, and if applicable, details of automation tools used in the process.	Methods (page 6)
Effect measures	12	Specify for each outcome the effect measure(s) (e.g. risk ratio, mean difference) used in the synthesis or presentation of results.	Methods (page 5 & 6)
Synthesis methods	13a	Describe the processes used to decide which studies were eligible for each synthesis (e.g. tabulating the study intervention characteristics and comparing against the planned groups for each synthesis (item #5)).	Methods (page 5 & 6)
	13b	Describe any methods required to prepare the data for presentation or synthesis, such as handling of missing summary statistics, or data conversions.	Methods (page 6)
	13c	Describe any methods used to tabulate or visually display results of individual studies and syntheses.	Methods (page 6)
	13d	Describe any methods used to synthesize results and provide a rationale for the choice(s). If meta-analysis was performed, describe the model(s), method(s) to identify the presence and extent of statistical heterogeneity, and software package(s) used.	Methods (page 6)
	13e	Describe any methods used to explore possible causes of heterogeneity among study results (e.g. subgroup analysis, meta-regression).	Methods (page 6)
	13f	Describe any sensitivity analyses conducted to assess robustness of the synthesized results.	Methods (page 6)
Reporting bias assessment	14	Describe any methods used to assess risk of bias due to missing results in a synthesis (arising from reporting biases).	N/A
Certainty assessment	15	Describe any methods used to assess certainty (or confidence) in the body of evidence for an outcome.	N/A
**RESULTS**			
Study selection	16a	Describe the results of the search and selection process, from the number of records identified in the search to the number of studies included in the review, ideally using a flow diagram.	Results page 7 and figure 1
	16b	Cite studies that might appear to meet the inclusion criteria, but which were excluded, and explain why they were excluded.	N/A
Study characteristics	17	Cite each included study and present its characteristics.	Supplementary tables 3 & 4
Risk of bias in studies	18	Present assessments of risk of bias for each included study.	Supplementary figures 1-3

Results of individual studies	19	For all outcomes, present, for each study: (a) summary statistics for each group (where appropriate) and (b) an effect estimate and its precision (e.g. confidence/credible interval), ideally using structured tables or plots.	N/A
Results of syntheses	20a	For each synthesis, briefly summarise the characteristics and risk of bias among contributing studies.	Supplementary tables 3-4
	20b	Present results of all statistical syntheses conducted. If meta-analysis was done, present for each the summary estimate and its precision (e.g. confidence/credible interval) and measures of statistical heterogeneity. If comparing groups, describe the direction of the effect.	Tables – 2-4; Supplementary Tables 5-13, Figures 3 & 4
	20c	Present results of all investigations of possible causes of heterogeneity among study results.	Supplementary Tables 6, 7, 9, 10, 11 & 13
	20d	Present results of all sensitivity analyses conducted to assess the robustness of the synthesized results.	Supplementary Tables 5, 8 &12
Reporting biases	21	Present assessments of risk of bias due to missing results (arising from reporting biases) for each synthesis assessed.	N/A
Certainty of evidence	22	Present assessments of certainty (or confidence) in the body of evidence for each outcome assessed.	N/A
**DISCUSSION**			
Discussion	23a	Provide a general interpretation of the results in the context of other evidence.	Discussion (pages 13-18)
	23b	Discuss any limitations of the evidence included in the review.	Discussion (page 16)
	23c	Discuss any limitations of the review processes used.	Discussion (page 16)

	23d	Discuss implications of the results for practice, policy, and future research.	Discussion page 17 % 18
**OTHER INFORMATION**			
Registration and protocol	24a	Provide registration information for the review, including register name and registration number, or state that the review was not registered.	Not registered
	24b	Indicate where the review protocol can be accessed, or state that a protocol was not prepared.	N/A
	24c	Describe and explain any amendments to information provided at registration or in the protocol.	N/A
Support	25	Describe sources of financial or non-financial support for the review, and the role of the funders or sponsors in the review.	Funding statement page 19
Competing interests	26	Declare any competing interests of review authors.	COI – page 19
Availability of data, code and other materials	27	Report which of the following are publicly available and where they can be found: template data collection forms; data extracted from included studies; data used for all analyses; analytic code; any other materials used in the review.	Data sharing statement page 19

Supplementary table 1: PRISMA reporting guidance

From: Page MJ, McKenzie JE, Bossuyt PM, Boutron I, Hoffmann TC, Mulrow CD, et al. The PRISMA 2020 statement: an updated guideline for reporting systematic reviews. BMJ 2021;372:n71. doi: 10.1136/bmj.n71. This work is licensed under CC BY 4.0. To view a copy of this license, visit https://creativecommons.org/licenses/by/4.0/

Meta-analysis Groups	Original study terms (Diagnosis and Symptoms) for grouping
**Anxiety and Related Symptoms**	Anxiety, Anxiety symptoms, Anxiety/Depression, Anxiety (mild), Anxiety disorder, Specific phobia, PTSD, Panic disorder, GAD, Agoraphobia, Social phobia
**Apathy**	Apathy
**Attentional Difficulties**	Attention, Attention deficits, Attentional difficulties, ADHD
**Autism Spectrum Disorders**	ASD, Autism
**Behavioral Symptoms**	Behavior problems, Behavior/personality change, Behavioral Concerns, Behavioral Problems, Behavioral abnormality, Behavioral change, Behavioral changes, Behavioral disturbance, Behavioral or personality change, Behavioral symptoms, Personality change, Personality changes, Withdrawal, Behavioral issues, Irritability and anger outbursts, Behavioral disorder, Behavioral disturbance, ODD
**Depressive Symptoms**	Depression, Depressive symptoms, Depressive symptoms and/or Anxiety symptoms, Depression (mild), Depressive disorder, Mild depression, Mood disorder
**Developmental Delay**	Developmental delay
**Disinhibition**	Aggression, Agitation, Disinhibition, Hyperactivity, Impulsivity, Increased Irritability, Irritability, Severe Disinhibition, Inattention/impulsivity, irritability, Hyperphagia, Hypersexual
**Disorientation**	Disorientation
**Eating Disorders**	Eating disorder
**Emotional Instability**	Emotional instability, Emotional lability, Emotionally reactive, Mood regulation disorder, Dysphoria, Pathologic tearfulness, Pathological laughter
**Impulse Control Disorders**	Impulse control disorder
**Learning Difficulties**	Learning Difficulties
**Manic Symptoms**	Manic symptoms, Hypomanic symptoms, Bipolar disorder, Euphoria, Euphoria/elation, Hypomania

**OCD Symptoms**	Obsessive-compulsive symptoms, OCD
**Other Mood Symptoms**	Mood problem, Elation, General Mood symptoms, Other mood symptoms, Mood disorder
**Personality Disorders**	Personality disorder
**Psychotic Symptoms**	Delusion, Hallucinations, Psychosis, Psychotic symptoms, Psychotic symptoms (mild), Psychotic symptoms (moderate), Thought problems, Blunted affect, Schizophrenia
**Sensory Hypersensitivities**	Hypersensitivity to sound, Hypersensitivity to light
**Sexual Disorders**	Sexual disorders
**Sleep disorders**	Sleep disorder
**Somatic Symptoms**	Somatization
**Substance Use Disorders**	Alcohol abuse, Drug Abuse
**Suicidality and Self-harm**	Suicidal Ideation/attempt, Suicidal thoughts, Suicidality, DSH (deliberate self-harm)
**Tics**	Motor tics, Tourette's

ASD; Autism Spectrum Disorders, OCD: Obsessive-Compulsive Disorder, NOS: Not Otherwise Specified

Supplementary table 2: Mapping of Original Study Diagnostic and Symptom Terms to Meta-analysis Symptom Groups

Ref	Study ID	Country	Study design	Etiology	Agent	Follow-up duration in months, Mean (SD) Where SD is not given – uniform interval * median where mean not given	Total sample size	Adult, child or mixed	Mean age (SD) *median where mean not given	Female (%)	Outcome	Definition of outcome	Number with outcome
1	Abboud et al 2022	USA	Case Series	Autoimmune	Mixed	8	33	Adult	47(20)	58	Mood disorder (depression)	NR	17
											Psychosis	NR	1
						18					Mood disorder (depression)	NR	19
											Psychosis	NR	2
2	Berkhout et al 2022	Australia	cohort	Infectious	HSV	12-84	18	Child	1(4.5)	50	ADHD	NR	1
											ASD	NR	**2**
											Behavioral Disturbance	NR	5
3	Butler et al 2024	UK	Cross sectional	Autoimmune	Mixed (38x NMDAR, 13x, Anti-LGI, 6x VGCK, 1x Anti-CASPR2,	84 (107)	112	Adult	50.1 (15.6)	66	Anxiety Symptoms	Self reported	91
											Mood Problems	Self reported	66

					15x ADEM, 6x STREAT, 33x Other)						Psychotic Symptoms	Self reported	9
											Aggression	Self reported	30
											Depression	PDSQ	43
											PTSD	PDSQ	36
											Eating disorder	PDSQ	14
											OCD	PDSQ	39
											Panic Disorder	PDSQ	27
											Psychosis	PDSQ	26
											Agoraphobia	PDSQ	27
											Social Phobia	PDSQ	45
											Alcohol abuse	PDSQ	14
											Drug abuse	PDSQ	4
											GAD	PDSQ	25
											Personality disorder	PDSQ	10
				Infectious	Mixed<br>167x HSV, 2x JE, 19x VZV, 8x Tick-borne, 3x		229				Anxiety Symptoms	Self reported	209
											Mood Problems	Self reported	181

					EBV, 30x Other)						Psychotic Symptoms	Self reported	25
											Aggression	Self reported	86
											Depression	PDSQ	91
											PTSD	PDSQ	74
											Eating disorder	PDSQ	20
											OCD	PDSQ	87
											Panic Disorder	PDSQ	53
											Psychosis	PDSQ	56
											Agoraphobia	PDSQ	69
											Social Phobia	PDSQ	104
											Alcohol abuse	PDSQ	39
											Drug abuse	PDSQ	6
											GAD	PDSQ	82
											Personality disorder	PDSQ	18
4	Diaz-Ariaz et al 2021	USA	Cross sectional	Autoimmune	Mixed	4(3)	375	Adult	44(17)	62	Depressive Disorder	BDI >= 4	198

5	Ercis et al 2023	Turkiye	Case Series	Mixed	Mixed	60	10	Adult	41.2(14.9)	67	Depressive symptoms	HSDR>8	1
											Delusion	NPI	0
											Hallucinations		0
											Euphoria/Elation		0
											Disinhibition		0
6	Gadian et al 2022	UK	Case Series	Autoimmune	NMDAR	36*	21	Child	8.92*	76	Behavioral Concerns	NR	6
					Seronegative		23		9.42*	43%	Behavioral Concerns	NR	12
7	Guasp et al 2022	Spain	Cohort	Autoimmune	NMDAR	4(2.6)	28	Mixed	27*(10.4)	79%	Depressive Disorder	HDRS>7	11
											Hypomania	YMRS>12	2
											Psychotic Symptoms (mild)	NR	8
											Psychotic Symptoms (moderate)	NR	3
											Schizophrenia	DSM-5	4
8	Harmon et al 2022	USA	Case Series	Autoimmune	GAD	>12	10	Child	9.9	NR	Psychotic Symptoms	NR	3
											Anxiety symptoms	NR	8
											Agitation	NR	8

											Impulsivity	NR	8
											Depressive Symptoms	NR	7
											OCD Symptoms	NR	7
											Sleep Disorders	NR	3
9	He at al 2023	China	Case Series	Autoimmune	NMDAR	5-6	5	Adult	22.4(2.89)	100	Emotional Instability	NR	2
10	Hirose et al 2022	Japan	Cohort	Autoimmune	NMDAR	58.5*	39	Mixed	20.5*	NR	Psychotic Symptoms	NR	8
							41				Personality Changes	NR	22
11	Huang et al 2022	China	Case Series	Autoimmune	Mixed	>6	13	Mixed	45(18)	31	Behavioral abnormality	NR	4
12	Huang et al 2022	China	Cohort	Autoimmune	LGI-1	21.5*(26.67)	43	Adult	57*(15.6)	35	Emotional instability	NR	3
13	Lee et al 2022	South Korea	Case Series	Autoimmune	Antibody Negative	>12	27	Child	10.1(4.4)	48	General Mood Symptoms	NR	14
											Hallucinations	NR	7
											Attentional Difficulties	NR	2
					NMDAR		9		11.4(5.6)	67	General Mood symptoms	NR	3

											Hallucination	NR	3
											Hyperactivity	NR	1
											Anxiety Symptoms	NR	1
											Paranoia	NR	1
14	Li et al 2021	China	Cohort	Autoimmune	NMDAR	10*	109	Child	6.8* (0.89)	57	ADHD	NR	9
15	Lin et al 2022	Taiwan	Case Series	Infectious	EV-A71	121.5 (58.1)	18	Child	2.1(2.0)	28	ADHD	DSM 5	6
											ODD		1
											Depression		1
											Motor tics		1
											Tourette's		1
											ASD		1
											Specific phobia		1
16	Lin et al 2021	China	Cohort	Autoimmune	Anti-GABABR	18*	31	Adult	52*	39	Delusions	NPI	1
											Hallucinations		6
											Depressive Symptoms		15

											Anxiety Symptoms		7
											Manic symptoms		5
											Apathy		21
											Disinhibition		4
											Irritability		4
17	Lino et al 2022	USA	Cohort	Infectious	West Nile Virus	84* (14.3)	20	Mixed	50.7*	45	Depressive disorder	Self reported	15
											Depressive disorder	CES >= 15	7
18	Marcus et al 2021	USA	Case Series	Autoimmune	NMDAR	26.35 (22.42)	11	Mixed	12.88 (6.13)	73	Behavioral symptoms	NR	6
											Depressive Symptoms		1
											Insomnia		1
											Impulsivity		1
				Mixed	NMDAR + HSV	20.63 (19.84)	6	Child	3.03 (5.81)	33	Behavioral Symptoms		3
19	Paralicova et al 2022	Slovakia	Case Series	Infectious	Tickborne	36*	17	Adult	48	53	Depressive Disorder	NR	1
											Bipolar Disorder		1
20	Ramesh et al 2023	India	Cohort	Autoimmune	Mixed	6*	31	Mixed	43.26 (21.43)	29	Behavioral Symptoms	NR	3

21	Ren et al 2021	China	Cohort	Autoimmune	NMDAR	48.5 (26.7)	20	Child	NR	NR	Disinhibited Behavior	NR	2
											ASD		1
											ADHD		1
22	Su et al 2021	China	Case Series	Infectious	Japanese Encephalitis	6*	7	Adult	48.89 (15.33)	NR	Depressive symptoms	NR	4
											Dysphoria		1
23	Sun et al 2023	China	Case series	Autoimmune	Anti-amphiphysin	10 (9.2)	10	Adult	52 (16.4)	60	Depressive symptoms	NR	3
											Anxiety symptoms		3
											Psychotic symptoms (mild)		1
											Irritability		1
											Apathy		1
24	Tellez-Martinet al 2023	Mexico	Case series	Autoimmune	NMDAR	43	15	Adult	32	53	Psychotic symptoms (mild)	NR	1
											Depressive symptoms		1
											Anxiety symptoms		1
											Suicidal thoughts		1

											Deliberate self harm		1
25	Warren et al 2021	Australia	Cohort	Autoimmune	NMDAR	14 (10.1)	30	Adult	37.5 (16.7)	60	Depressive disorder	Clinical assessment	4
											Psychotic disorder		3
26	Wilkinson-Smith et al 2022	USA	Cohort	Autoimmune	NMDAR	31.83 (9.02)	22	Child	8.78 (5.02)	65	Hyperactivity	BASC	8
											Aggression		7
											Conduct problems		4
											Anxiety symptoms		10
											Depressive symptoms		8
											Somatization		8
											Atypicality		7
											Withdrawal		6
											Attentional difficulties		11
											Behavioral symptoms		8
27	Wu et al 2023a	China	Cohort	Autoimmune	NMDAR	46	58	Adult	29	53.6	Anxiety disorder	GAD >= 7	6
											Depressive disorder	PHQ-9 >= 5	5

28	Wu et al 2021	Taiwan	Case series	Autoimmune	NMDAR	69.6 (39.6)	10	Adult	3.6	90.0	Hallucinations	Notes review	1
											Aggression		1
29	Wu et al 2023b	Taiwan	Cross sectional	Autoimmune	NMDAR	42	10	Child	7.9	80.0	ADHD	NR	1
											Mood disorder		1
30	Yokota et al 2023	Japan	Cross sectional	Autoimmune	Mixed<br><br>Definite autoimmune limbic encephalitis (n = 2), definite NMDARE (n = 10), definite Bickerstaff brainstem encephalitis (n = 1), definite AE (n = 3), probable Hashimoto encephalopathy (n = 1), and autoanti-body-negative but probable AE (n = 2)	63	21	Mixed	26	71.4	Mood disorder	NR	1
											Personality change		3
31	Zeng et al 2020	China	Case series	Autoimmune	Anti-GABAR	16.1 (4.4)	7	Adult	44.7 (14.1)	42.8	Psychosis	NR	1
32	Wang et al 2019	China	Case-Control	Autoimmune	NMDAR	9.5	16	Mixed	29.7	68.8	Psychotic symptoms	NPI	0
33	Tong et al 2020	China	Case Series	Autoimmune	Mixed (seronegative n =18, NMDAR n =10)	12-36	28	Child	NR	32.1	Personality change	NR	1

34	Sejvar et al 2007	Bangladesh	Case series	Infectious	Nipah Virus	14	17	Mixed	14.5	NR	Behavioral problems	Carer/self-report (Functional self-assessment rating scale)	6
											Depressive symptoms		10
35	Prakash Gangwar et al 2020	India	Case series	Infectious	Orientia tsutsugamushi	5.4	146	Child	7.2 (4.2)	46.6	Behavioral difficulties	Liverpool outcome score	74
36	Ooi et al 2008	Malaysia	Case series	Infectious	Japanese Encephalitis	52.9	86	Child	NR	42.7	Hyperactivity	Clinical assessment using self-developed functional outcome score	2
											Personality change/psycho Behavioral problems		7
											Blunted affect		1
37	Von Rhein et al 2016	Germany	Case series	Autoimmune	Seronegative	18 (17)	25	Adult	48 (15)	28.5	Depressive symptoms	BDI >10	9
38	Sola-Valls et al 2020	Spain	Case-control	Autoimmune	LGI-1	87	36	Adult	74	36.1	Emotional lability	Self/carer report	6
39	Schmidt et al 2011	Switzerland	Case series	Mixed	Idiopathic	24*	32	Adult	47.2 (16.6)	43.6	Depression	GDS	4
40	Sarkis et al 2014	USA	Case series	Autoimmune	NMDAR	36	5	Adult	43.2	60.0	Anxiety disorder	Chart review	1
											Depressive disorder		0
											Impulse control disorder		0

											Psychosis		0
											Suicidality/sel f-harm		0
					Seronegative	36	7		48.7	85.7	Anxiety disorder		1
											Depressive disorder		2
											Impulse control disorder		1
											Psychosis		0
											Suicidality/sel f-harm		0
41	Sarkari et al 2012	India	Case series	Infectious	Japanese Encephalitis	168*	453	Adult	NR	NR	Depressive symptoms	Clinical assessment /self-report	6
											Euphoria		0
											Emotional lability		9
											Attentional difficulties		11
											Hyperactivity		10
											Aggression		10
											Hallucinations	Self/carer report	0
42	Rao et al 2017	USA	Case series	Mixed	Mixed (HSV n = 9,	15.6	49	Child	6.8	49.0	Behavioral problem		15

					Enterovirus n = 6, Influenza n = 3, Idiopathic n = 31)						Anxiety/depression		18
43	Ng et al 2004	Singapore	Case series	Infectious	Nipah virus	24*	9	Adult	40.8 (10.9)	11.1	Aggression	SCAN	1
											Irritability		1
											OCD symptoms		0
											Anxiety		0
											Depression		5
											Agitation		0
											Manic symptoms		0
											Personality change		2
44	Nair et al 2019	India	Case series	Autoimmune	NMDAR	12.3	6	Child	6.5	83.3	Aggression	NR	1
45	Nagappa et al 2016	India	Case series	Autoimmune	NMDAR	10.3 (6.7)	9	Child	NR	88.8	Behavior/personality change	Notes review	8
											Hyperactivity		6
											Apathy		2
											Eating disorders		3

											Disinhibition		5
46	Murray et al 2007	USA	Cohort	Infectious	WNV	12*	38	Adult and child	55.0	28.9	Depression	Self/carer report	13
											Irritability		12
											Personality change		18
47	McKeon et al 2016	Australia	Case control	Autoimmune	NMDAR	23.1	7	Adult	26.42 (8.54)	85.7	Anxiety symptoms	Self-report	4
											Emotional lability		5
											Psychotic symptoms		1
											Personality change		2
											Disinhibition		1
											Depressive symptoms		4
48	McGrath et al 1997	New Zealand	Case control	Infectious	HSV	19.2	29	Mixed	45	NR	OCD symptoms	Self-report	2
											Depressive symptoms		5
											Behavioral/personality change		14
											Suicidality		2
											Eating disorders		1

49	Maureille et al 2019	France	Case series	Autoimmune	Anti-GABABR	24*	6	Adult	63.0	0%	Behavioral disorder	Patient records	1
											Depressive symptoms		4
50	Mailles et al 2012	France	Case series	Infectious	HSV	31	33	Mixed	58.0	44.2	Anxiety symptoms	Glasgow outcome score	1
											Depressive symptoms		4
											Disinhibition		5
											Irritability		6
											Disorientation		7
					VZV		14		63.0	20.0	Anxiety symptoms		0
											Depressive symptoms		1
											Disinhibition		0
											Irritability		1
											Disorientation		3
					Tuberculosis		9		64.0	50.0	Anxiety symptoms		0
											Depressive symptoms		0
											Disinhibition		0

											Irritability		2
											Disorientation		0
					Mixed (Listeria monocytogenes (n = 4), tick-borne encephalitis (n = 3), Mycoplasma pneumoniae (n = 2), Epstein-Barr virus (n = 2), cytomegalovirus (n = 2), enterovirus (n = 2), Legionella pneumophila (n = 1), influenza A (n = 1), Borrelia burgdorferi (n 5=1), Rickettsia coronii (n = 1), Francisella tularensis (n = 1), Cryptococcus neoformans (n = 1), and Toscana virus (n = 2).		21		51.0	26.1	Anxiety symptoms		0
											Depressive symptoms		0
											Disinhibition		0
											Irritability		2
											Disorientation		0
				Mixed	Idiopathic		61		43.0	41.7	Anxiety symptoms		3
											Depressive symptoms		4

											Disinhibition		1
											Irritability		8
											Disorientation		7
51	Berlit et al 1988	Germany	Case series	Infectious	Mixed (HSV n =7, VZV n=2, Rubella n = 2, Adenovirus n = 1, idiopathic n = 11)	48	23	NR	NR	42.4	Depressive symptoms	Self-report	6
52	Blum et al 2020	USA	Case series	Infectious	NMDAR	52.8 (37.2)	61	Mixed	33.6 (12.8)	90.1	Emotional lability	Self-report	34
53	Cainelli et al 2019	Italy	Case series	Autoimmune	NMDAR	35	5	Child	9.2	60.0	Aggression	SAFA, CBCL, K-Sads-PL	1
											Behavioral or personality change		3
											Disinhibition		3
											Irritability		1
											Obsessive-compulsive symptoms		1
											Hyperactivity		1
											Apathy		1
											Emotional lability		1

											Depressive symptoms		3
											Impulsivity		2
54	Caparros-Lefebvre et al 1996	France	Case series	Infectious	HSV	Range 6-48	11	Adult	51.3	18.2	Irritability	Clinical assessment	7
											Anxiety disorder	Symptom scales for DSM-3-R	5
											Panic disorder		3
											OCD		0
											Apathy		1
											Sexual disorder		1
											Eating disorder		1
											Depressive symptoms	HAM-D	2
											Emotional lability	Allman scale	7
55	De Bruijn et al 2018	Netherlands	Case series	Autoimmune	NMDAR	31	22	Child	14	77.2	Impulsivity	Self/carer report	2
											Irritability		4
											OCD symptoms		1
											Anxiety symptoms		3

											Depressive symptoms		1
											Emotional lability		1
											Attentional difficulties		2
											Disinhibition		7
											Apathy		5
56	Domachowske et al 1996	USA	Case series	Infectious	EBV	Range 24-108	10	Child	8.6	50	Hyperphagia	Patient records	2
											Hypersexuality		1
											Hyperactivity		1
											Disinhibition		1
57	Engman et al 2012	Sweden	Case control	Infectious	TBE	Range 12-18	8	Child	8.4	62.5	Behavioral changes	Clinical interview and FTFQ	2
											Attentional problems		2
											Hyperactivity		2
											Irritability		4
58	Fazekas 2006 et al	Austria	Case series	Infectious	HSV	62.4 (37.2)	26	Adult	49.3	38.4	Depressive disorder	WHO-5 <13	10
59	Gordon-Lipkin et al	Case series	USA	Autoimmune	NMDAR	43.2	6	Adult	31.9	100	Depressive symptoms		0

	2017 Adults										Agitation	Structured telephone survey	0
											Disinhibition		0
											Emotional lability		3
59	Gordon-Lipkin et al 2017 Children	Case series	USA	Autoimmune	NMDAR	28.8	4	Child	5.1	40	Depressive symptoms	Structured telephone survey	0
											Agitation		0
											Disinhibition		0
											Emotional lability		0
60	Fowler et al 2013	Sweden	Case series	Infectious	TBE	50.4	42	Child	10.8	40.5	Hypersensitivity to sound	RPQ	15
											Irritability		10
											Depressive symptoms		12
											Frustration		7
											Psychotic symptoms		14
											Hypersensitivity to light		11
											Hyperactivity		10
											Personality change		10

61	Griffiths et al 2014	Nepal	Case series	Infectious	Japanese encephalitis	8	5	Child	9	37.5	Behavioral disturbance	Clinical assessment	5
				Mixed	NR		16				Behavioral disturbance		16
62	Hokkanen et al 1997	Finland	Case series	Mixed	HSV n = 5<br>Adenovirus n = 1<br>Puumala virus n = 1<br>Unknown n = 5	36.5	12	Adult	47.7	25	Panic disorder	NR	1
											Anxiety disorder		1
											Bipolar disorder		1
											Irritability		2
											Depressive disorder		4
63	Ho et al 2018	Hong Kong	Case series	Autoimmune	NMDAR	20.5	14	Child	12	83	Irritability	Medical records using standardized questionnaire	2
											Disinhibition		1
											Emotional lability		1
											Behavioral problems		2
64	Binks et al 2024	UK	Case series	Autoimmune	LGI-1	41	44	Adult	64	33	Anxiety	HADS-A >7	11
											Depression	HADS-D >7	19
											Pathological laughter	Clinical assessment	11
65	Harris et al 2020	UK	Case control	Infectious	HSV	12*	30	Adult	48.37 (13.8)	66	Depressive disorder	BDI >=14	15

											Anxiety disorder	BAI >=8	14
				Infectious	Mixed (Not HSV)		10		49.7 (16.17)	50	Depressive disorder	BDI >=14	2
											Anxiety disorder	BAI >=8	5
				Autoimmune	Mixed		14		44.21 (19.35)	50	Depressive disorder	BDI >=14	6
											Anxiety disorder	BAI >=8	5
				Mixed	NR		27		50 (12.56)	59.3	Depressive disorder	BDI >=14	8
											Anxiety disorder	BAI >=8	10
66	Do Los Reyes et al 2008	USA	Case series	Infectious	La Crosse	120*	5	Child	7	20	ADHD	NR	2
											Severe disinhibition		1
67	Michaeli et al 2014	Israel	Case series	Infectious	Mixed (Enterovirus n = 9, HSV n = 6, Other n =8, NR n = 23)	69.6 (36.96)	46	Child	4.88	39.1	ADHD	DSM-5	23
											Tic Disorder		7
											Behavioral problems	Conners' Parent Rating Scales–Revised	24
68	Ogata et al 2017	Japan	Cohort	Infectious	HHV-6	55.0	121	Mixed	NR	33.6	Personality change	NR	3
											Behavioral abnormality		7

											Disorientation		8
69	Arino et al 2020	Spain	Case control	Autoimmune	NMDAR	6	18	Mixed	28.9	88.8	Irritability	Clinical assessment	5
											Apathy		2
											Emotional lability		3
											Depressive symptoms		1
70	Basheer et al 2017	India	Case series	Autoimmune	NMDAR	20.8 (12.6)	20	Child	10.4 (4.9)	75.0	Irritability/Anger outbursts	BPRS-C	4
71	Brenton et al 2016	USA	Case series	Autoimmune	NMDAR	12	10	Child	13	80.0	Behavioral abnormalities	NR	4
72	Chanvanichtrakool et al 2017	Thailand	Case series	Autoimmune	NMDAR	28.4 (13.1)	13	Child	12 (3.3)	69.2	OCD	Notes review	1
											Mood disorder		3
73	Chou et al 2013	Taiwan	Cross sectional	Autoimmune	Mixed (Anti-GAD n =3, anti-amphiphysin n = 2, seronegative n = 5)	67.2	10	Child	11.5 (4.1)	40.0	Hallucinations	Notes review	2
											Behavioral change		3
											Suicidality		1
											Hyperactivity		§
											Irritability		§
74	Pretkalnina et al 2024	Latvia	Case series	Autoimmune	Mixed (NMDAR = 4, GAD-65 = 1, ZIC-4 = 1,	23 (21.9)	18	Child	8	44.4	Learning difficulties	NR	6

					Seronegative =12)								
75	Chen et al 2024	Spain	Case series	Autoimmune	NMDAR	85.1 (18)	68	Child	9.7	68.4	Behavioral problems	Liverpool outcome score	19
											Inattention/impulsivity		10
											Mood problems		7
											Anxiety		3
											ASD		2
											Depression		1
											Unspecified psychiatric disorder		1
											ADHD		2
76	Morgan et al 2024	USA	Case series	Autoimmune	NMDAR	17.7	18	Mixed	28	76.3	Depression	PHQ-9	10
											Anxiety	GAD-7	8
77	Griška et al 2024	Lithuania	Case control	Infectious	TBE	17.7	61	Adult	54.3 (15.6)	40.9	Irritability	Self-report	18
											Emotional instability		7
78	Bergman et al 2024	Sweden	Cohort	Mixed	Unknown, n = 18	41	40	Child	4.3	52.5	Anxiety/depression	CBCL	2 (of 40)

					Tick Borne encephalitis, n = 10<br>Rotavirus n = 7<br>Enterovirus = 6,<br>Varicella Zoster Virus = 4,<br>Influenza A/B virus n = 3<br>Epstein Barr Virus n = 3<br>HSV1 + NMDAr, n 2<br>VZV + NMDAr, n = 1<br>Coronavirus, n = 1<br>Adenovirus, n= 1<br>Multiple aetiologies , n = 3						Disinhibition		5 (of 41)
											Depression		1 (of 15)
											Emotionally reactive		4 (of 25)
											Aggression		2 of (40)
											Attentional difficulties		4 (of 40)
											Thought disorder		1 (of 15)
79	Muñoz-Lopetegi et al 2024	Spain	Cohort	Autoimmune	LGI-1	15	20	Adult	65	50	Behavioral or psychiatric complaints	Structured questionnaire	
											Depression (mild)	HDRS (8-16)	8
											Depression with psychotic features	Positive PANSS and HDRS score	1
80	Dwibedi et al 2024	India	Case series	Autoimmune	NMDAR	6*	32	Child	7.0 (5.2)	56.7	Behavioral abnormality	Clinical assessment	10
				Infectious	HSV	6*	206		6.0 (4.4)	47.5			37

81	Mangioris et al 2024	USA	Case series	Autoimmune	Seronegative	36	45	Adult	61	46.6	Attention deficits	Clinical assessment	17
											Behavior/personality change		5
											Psychosis		1
											Anxiety		5
											Mood		3
82	Madani et al 2024	Canada	Case series	Autoimmune	Seronegative	12.2 (3.3)	56	Children	7 (5.5)	NR	Behavioral changes	Clinical assessment	9
83	Dumez et al 2024	France	Case series	Autoimmune	NMDAR (Post HSE)	12	13	Adult and child	19	46.2	Behavioral disorder	Clinical assessment	8
84	Benoit et al 2023	France	Case series	Autoimmune	CASPR-2	58.8	48	Adult	64	2.0	Behavioral disorder	Medical records	25
											Mood disorder		21
85	Finke et al 2012	Germany	Case series	Autoimmune	NMDAR	43.0	9	Adult	28.4	88.8	Attentional difficulties	Self-report	1
											Distractibility		1
86	Heine et al 2021	Germany	Cohort	Autoimmune	NMDAR	27.6	40	Adult and child	28.5 (7.2)	87.5	Depressive symptoms	BDI-2 (mild or worse)	13
											Anxiety symptoms	BAI (mild or worse)	24
87	Byrne et al 2014	Ireland	Case series	Autoimmune	NMDAR	24	5	Child	6.8 (2.59)	60	Behavioral issues	NR	1
88	Do Valle et al 2019	Brazil	Case series	Autoimmune	NMDAR	36	9	Child	5.0	66.7	Irritability	NR	1

89	Raja et al 2021 - adult	India	Case series	Autoimmune	NMDAR	24*	5	Adult	29.8	40	Behavioral problems	NR	0
89	Raja et al 2021 - children	India	Case series	Autoimmune	NMDAR	24*	16	Child	10.5	93.4	Behavioral problems	NR	1
90	Liu et al 2021	China	Case series	Autoimmune	Autoimmune after Japanese Encephalitis (idiopathic n=2, NMDAR=2, GABA-B =2)	12	5	Child	10.4 (1.85)	40	Emotional instability	NR	1
91	Wang et al 2017	China	Case series	Autoimmune	NMDAR	16.1	51	Child	8	58.8	Mood regulation disorder	NR	2
92	Yeshokumar et al 2022	USA	Case series	Autoimmune	NMDAR	48 (28.8)	39	Mixed	19.1 (16.9)	73.2	Emotional lability	Structured clinical interview	19
93	Thakolwiboon et al 2025	USA	Case series	Autoimmune	Overall mixed cohort	44	119	Adult	57 (22.96)	43	Personality changes	DSM-5	7
											Other mood symptoms		6
											Anxiety		13
											Depression		27
											Psychosis		5
											Suicidal Ideation/attempt		1
					NMDAR	44	15	Adult	NR	NR	Anxiety		1

											Depression		2
											Psychosis		0
					LGI-1	44	31	Adult	NR	NR	Anxiety		0
											Depression		7
											Psychosis		0
					GAD-65	44	20	Adult	NR	NR	Anxiety		3
											Depression		8
											Psychosis		2
					Seronegative	44	11	Adult	NR	NR	Anxiety		1
											Depression		3
											Psychosis		1
94	Liu et al 2019	China	Case series	Autoimmune	NMDAR	48*	37	Mixed	NR	NR	Irritability	Neuropsychiatric inventory	0
											Aggression		0
											Hallucinations		0
											Delusions		0
											Anxiety		0

											Elation		0
											Apathy		0
											Disinhibition		0
											Depression		0
95	Galioto et al 2023	USA	Case series	Autoimmune	LGI-1	38.5	10	Adult	67.5	40	Depression	BDI-2 >1.5SD from normative	4
											Anxiety	BAI >1.5 SD from normative	6
101	Argyropoulos et al (2020)	UK	Case series	Autoimmune	Mixed	64.9	38	Adut	63.1	32	Pathologic tearfulness	Clinical interview	19

Supplementary table 3: Study characteristics

HSV – Herpes Simplex Virus, **JE** – Japanese Encephalitis, **VZV** – Varicella Zoster Virus, **EBV** – Epstein-Barr Virus, **EV-A71** – Enterovirus A71, **TBE** – Tick-borne Encephalitis, **WNV** – West Nile Virus, **NMDAR** – Anti-N-methyl-D-aspartate receptor, **LGI-1** – Leucine-rich glioma-inactivated 1, **CASPR2** – Contactin-associated protein-like 2, **GAD** – Glutamic acid decarboxylase, **GAD-65** – Glutamic acid decarboxylase 65 isoform, **GABABR** – Gamma-aminobutyric acid type B receptor, **AE** – Autoimmune Encephalitis, **ADEM** – Acute Disseminated Encephalomyelitis, **STREAT** – Steroid-responsive Encephalopathy Associated with Autoimmune Thyroiditis, **ZIC-4** – Zinc finger protein 4, **PANSS** – Positive and Negative Syndrome Scale, **BDI** – Beck Depression Inventory, **BDI-2** – Beck Depression Inventory, Second Edition, **BAI** – Beck Anxiety Inventory, **PDSQ** – Psychiatric Diagnostic Screening Questionnaire, **HDRS** – Hamilton Depression Rating Scale, **HAM-D / HAMD** – Hamilton Rating Scale for Depression, **HAM-A** – Hamilton Anxiety Rating Scale, **YMRS** – Young Mania Rating Scale, **PHQ-9** – Patient Health Questionnaire-9, **GAD-7** – Generalized Anxiety Disorder 7-item scale, **CES** – Center for Epidemiologic Studies Depression Scale, **Zung Depression Scale / Zung Anxiety Scale** – Self-rated depression/anxiety measures, **NPI** – Neuropsychiatric Inventory, **BASC** – Behavior Assessment System for Children, **HADS / HADS-A / HADS-D** – Hospital Anxiety and Depression Scale (Total, Anxiety, Depression), **DSM-5** – Diagnostic and Statistical Manual of Mental Disorders, Fifth Edition, **DSM-3-R** – Diagnostic and Statistical Manual of Mental Disorders, Third Edition, Revised, **RPQ** – Rivermead Post-Concussion Symptoms Questionnaire, **SAFA** – Psychopathology Assessment Schedule for Adolescents and Children, **CBCL** – Child Behavior Checklist, **K-SADS-PL** – Schedule for Affective Disorders and Schizophrenia for School-Age Children – Present and Lifetime Version, **BPRS-C** – Brief Psychiatric Rating Scale for Children, **EBIQ** – European Brain Injury Questionnaire, **SCAN** – Schedules for Clinical Assessment in Neuropsychiatry.

Reference	Study	Country	Study design	Cohort (HC vs cases (including agent)	Follow-up duration in months, Mean (SD) Where SD is not given – uniform interval * median where mean not given	Total sample size	Adult, child or mixed	Mean age (SD) *median where mean not given	Female (%)	Outcome	Definition of outcome	Severity score (median (SD) unless stated)
96	Chen et al 2022	China	Case control	23 Health controls vs 21 with NMDAR encephalitis	6	44	Adults	Controls-26.43 (5.22)<br>Cases-27.48 (9.49)	64	Anxiety	Self-Rating Anxiety Scale	Control 22.09 [mean] (2.54)<br>Case-23.33 [mean] (2.85)
										Depression	Self-Rating Depression Scale	Control-21.91 [mean] (3.18)<br>Case- 22.61 [mean] (2.58)
5	Ercis (2023)	Turkey	Case Series	Mixed	60	10	Mixed	41.2 (14.89)	66.7	Agitation/Aggression	Neuropsychiatric Inventory	1.1 [mean]
										Dysphoria/Depression	Neuropsychiatric Inventory	0.4 [mean]
										Anxiety	Neuropsychiatric Inventory	0.3 [mean]
										Apathy/Indifference	Neuropsychiatric Inventory	2.2 [mean]
										Irritability/Lability	Neuropsychiatric Inventory	1.6 [mean]
										Nighttime Behavior	Neuropsychiatric Inventory	0.5 [mean]

95	Galioto (2023)	USA	Case Series	10- LGI-1 cases<br>20 Controls	9	30	Adults	Controls- 67 (7.1)<br>Cases- 64.2 [mean] (10.3)	46.7- control<br><br>40- cases	Depression	Beck Depression Inventory, Second Edition	Control- 12.9 [mean] (9.79)<br><br>Cases- 12.5 [mean] (10.9)
										Anxiety	Beck Anxiety Inventory	Control- 7.9 [mean] (6.4)<br><br>Cases- 7.6 [mean] (5.6)
7	Guasp (2022)	Spain	Cohort	28 NMDAR Encephalitis<br><br>27 Schizophrenia spectrum disorders<br><br>27 healthy controls	NMDAR: 16 months (IQR 15–19)<br><br>HC: 16 months (15–19 range)<br><br>Schizophrenia: 40 months (IQR 16-130)	82	Mixed	NMDAR 27<br>Schizophrenia 20<br>Health controls 23	NMDAR-78.6<br><br>Schizophrenia- 55.6<br><br>HC- 77.8	Psychosis symptoms	PANSS Positive	NMDAR- 9 (2.22)<br>SCZ-12 (9-17)<br>HC-7 (7-7)
											PANSS Negative	NMDAR-15 (10.37)<br>SCZ-18 (13-21)<br>HC-7 (7-7)
											PANSS General	NMDAR- 30 (8.88)<br>SCZ-28 (23-39)<br>HC- 16 (16-17)
											PANSS Total	NMDAR-55 (16.3)<br>SCZ-60 (45-78)<br>HC- 30 (30-32)
										Depression or Anxiety	HDRS	NMDAR-6 (3.7)<br>SCZ- 6 (3-12)<br>HC- 0 (0-1)
										Manic symptoms	YMRS	NMDAR-4 [(3.7)<br>SCZ-2 (0-5)<br>HC- 0 (0-0)
26	Wikinson-Smith (2022)	USA	Case series	23 NMDAR encephalitis	18.33 (6.44)	23	Children	8.78 (5.02)	65.2	Hyperactivity	BASC	93 [mean]
										Aggression	BASC	93.7 [mean]
										Conduct problems	BASC	99.4 [mean]
										Anxiety	BASC	92.9 [mean]

										symptoms		
										Depressive symptoms	BASC	89.4 [mean]
										Somatization	BASC	92.5 [mean]
										Atypicality	BASC	91.6 [mean]
										Withdrawal	BASC	96.5 [mean]
										Attentional difficulties	BASC	93.7 [mean]
										Behavioral symptoms	BASC	91.5 [mean]
97	Yeshokumar (2021)	UK	Case Series	266 Mixed Encephalitis	473.04	266	Mixed	50.26 (14.25)	67.3	Impulsivity	EBIQ	1.75 [mean] (0.48)
										Depression	EBIQ	1.85 [mean] (.51)
86	Heine (2021)	Germany	Case Series	40 NMDAR Encephalitis	27.6 (median)	40	Adults and Children	28.5 (7.2)	87.8	Depression	Beck depression	8 (0-24)
										Anxiety	Beck Anxiety (mild or worse)	12 (0-27)
85	Finke (2012)	Germany	Case Series	9 NMDAR encephalitis	43 (median)	9	Adults	28.4 (21-44)	88.9	Depressive symptoms	HAMD	2.9 (2.2)
98	Wang (2016)	China	Case series	39 NMDAR encephalitis	12 (median)	51	Adults and children	Median age 21.6	62.7	Depressive symptoms	Zung Depression Scale	43 (mean)
										Anxiety symptoms	Zung Anxiety Scale	40 (mean)

32	Wang (2019)	China	Case-control	16 NMDAR encephalitis<br><br>15 healthy controls	9.5 (median)	31	Adults and children	Cases- 29.7, controls- 30.3 (7.7)	Cases- 68.8, controls 66.7	Anxiety symptoms	Self-rating anxiety scale	Cases- mean: 25.64, controls- mean (23.87 (3.04)
										Depressive symptoms	Self-rating depression scale	Cases- mean: 26.21, control- mean: 23.53(3.83)
38	Sola-Valls (2020)	Spain	Case-control	36 LGI-1 encephalitis<br><br>23 healthy controls	Median follow up 87 months	60	Adults	Median age cases- 27	36.1% cases	Depressive and or anxiety symptoms	HADS	Case: mean: 5.5<br><br>Control mean: 2
47	McKeon (2016)	Australia	Case-control	7 NMDAR encephalitis<br><br>14 healthy controls	Follow up range 7-41 months	21	Adults	26.42 (8.54) cases, 25.81 (7.71) controls	85.7% cases, 71.4% controls	Anxiety symptoms	HADS	Case- Mean (SD) 5.35 (3.87)<br>Control- Mean (SD): 5.35 (3.87)
										Depressive symptoms	HADS	Case-Mean (SD) 1.78 (1.47)<br>Control- Mean (SD): 1.78 (1.47)
99	Cai (2020)	China	Case-control	17 NMDAR encephalitis<br><br>18 healthy controls	20.3 (14)	35	Not specified	28.59 (11.07) cases, 27.5 (7.38) controls	47.1 cases, 50% controls	Anxiety symptoms	HAMA	Case- Mean (SD) 4.12 (3.44)<br>Control- Mean: 1.17 (1.62)
										Depressive symptoms	HAMD	Case- Mean (SD) 5.59 (6.78)<br>Control- Mean (SD): 0.78 (1.17)
58	Fazekas (2006)	Austria	Case series	26 HSV encephalitis	62.4 (37.2)	26	Adult	49.3 (15)	38.5	Depressive symptoms	WHO-5	Mean (SD)<br>13.4 (5.8)
100	Meyding -Lamadé (2019)	Germany	Case series	32 HSV encephalitis	6	32	Adult	60.1 (13.6)	44.7	Depressive symptoms	HADS	Mean (SD)<br>7.2 (4.7)
										Anxiety symptoms	HADS	Mean (SD) 7.5 (4.2)

65	Harris (2020)	United Kingdom	Cohort	30 infectious- HSV, 10 infectious- mixed, 14<br><br>Autoimmune- mixed, 27- unknown<br><br>70 healthy controls	Minimum follow up 12 months for all patients	81	Adult	Infectious HSV: 48.37 (13.8),<br><br>Infective- mixed: 49.7 (16.17),<br><br>Autoimmune- mixed: 44.21 (19.35), Unknown- 50 (12.56)<br><br>Controls 50.80 (16.91)	Infectious HSV: 66.7% Infective- mixed: 50%<br><br>Autoimmune- mixed: 50%<br><br>Unknown: 59.3%<br><br>Healthy controls 54.0%	Depressive symptoms	BDI	Mean (SD)<br><br>Infective HSV- 13.81 (8.13)<br>Infective- mixed- 10.3(14.63)<br><br>Autoimmune-mixed- 15 (9.25)<br>Other- 12.2 (11.5)<br><br>Controls - 5.48 (5.37)
										Anxiety symptoms	BAI	Mean (SD)<br><br>Infective HSV- 10.04 (9.51)<br><br>Infective- mixed- 9.6 (10.43)<br><br>Autoimmune-mixed- 10.4 (9.67)<br><br>Other- 9.36 (9.38)<br><br>Controls 4.36 (4.64)
69	Ariño et al 2020	Spain	Case-control	14 NMDAR encephalitis<br><br>17 controls	Median 6	31	Adult	29.3- cases 24.6- controls	80.7 cases, 76.5% controls	Depressive symptoms	HAMD	Case- Mean (SD) 8 (4.4)<br>Control- Mean (SD)- 0.9 (1.9)
										Manic symptoms	YMRS	Case- Mean (SD) 5.3 (4.9)<br>Control- Mean (SD) 0.6 (1.2)
										Psychotic symptoms	PANSS	Case- Mean (SD) 53.1 (11.8)<br>Control- Mean (SD) 30.8 (1.6)

69	Ariño et al 2020	Spain	Case-control	4 NMDAR encephalitis<br><br>4 controls	Median 6	8	Child	13- cases<br><br>15.25- controls	100% cases, 100% controls	Depressive symptoms	HAMD	Case-Mean (SD) 1.5 (2.1)<br>Control- 0
										Manic symptoms	YMRS	Mean (SD) 2.8 (2.6)<br>Control-0
										Psychotic symptoms	PANSS	Mean (SD)<br>36.3 (5) Control-0
70	Basheer et al 2017	India	Case series	21 NMDAR encephalitis	20.8 (+/- 12.6)	21	Children	10.4 (+/- 4.9)	76.2	Psychiatric symptoms as rated on Brief Psychiatric Rating Scale for Children (BPRS-C)	BPRS-C	Mean BPRSC 0.6 (+/- 1.2)

Supplementary table 4: Study characteristics of studies reporting severity

NMDAR – Anti-N-methyl-D-aspartate receptor, **LGI-1** – Leucine-rich glioma-inactivated 1, **GABABR** – Gamma-aminobutyric acid type B receptor, **CASPR2** – Contactin-associated protein-like 2, **BDI** – Beck Depression Inventory, **BAI** – Beck Anxiety Inventory, **HAMD** – Hamilton Depression Rating Scale, **YMRS** – Young Mania Rating Scale, **Zung Depression Scale** – Zung Self-Rating Depression Scale, **Zung Anxiety Scale** – Zung Self-Rating Anxiety Scale, **HADS** – Hospital Anxiety and Depression Scale, **HADS-A** – Hospital Anxiety and Depression Scale – Anxiety subscale, **HADS-D** – Hospital Anxiety and Depression Scale – Depression subscale, **PANSS** – Positive and Negative Syndrome Scale, **BASC** – Behavior Assessment System for Children, **EBIQ** – European Brain Injury Questionnaire, **DSM-5** – Diagnostic and Statistical Manual of Mental Disorders, 5th Edition, **BPRS-C** – Brief Psychiatric Rating Scale for Children, **Self-Rating Anxiety Scale** – Self-report anxiety assessment tool, **Self-Rating Depression Scale** – Self-report depression assessment tool, **WHO-5** – World Health Organization-Five Well-Being Index, **NR** – Not Reported.

Adults only						
**Symptom**	**Prevalence (%)**	**95% Lower CI (%)**	**95% Higher CI (%)**	**Number of patients**	**I2**	**Number of cohorts**
Psychotic Symptoms	8.7	5.4	13.8	1188	71.5	19
Depressive Symptoms	29.3	23.7	35.6	1986	81.1	40
Attentional Difficulties	14.6	3.6	44.3	507	92.5	3
Behavioral Symptoms	20.4	8.0	43.1	226	81.6	7
Anxiety and Related Symptoms	28.0	17.7	41.3	888	88.2	24
Other Mood Symptoms	40.3	5.2	89.2	460	99.0	3
Disinhibition	18.1	9.6	31.6	958	89.6	12
Eating Disorders	10.1	7.3	13.9	352	3.5	3
OCD Symptoms	36.3	31.4	41.5	361	0.0	4
Substance Use Disorders	15.5	11.7	20.3	341	14.3	2
Personality Disorders	8.2	5.7	11.7	341	0.0	2
Emotional Instability	26.3	16.2	39.7	740	82.8	12
Apathy	23.2	5.8	59.8	66	78.0	4
Suicidality and Self-Harm	3.7	1.0	12.7	144	20.5	4
Impulse Control Disorders	14.7	2.9	49.8	10	0.0	2
**Children only**						
**Symptom**	**Prevalence**	**95% Lower CI**	**95% Higher CI**	**Number of patients**	**I2**	**Number of cohorts**
Psychotic Symptoms	16.3	8.9	28.0	219	51.5	8
Depressive Symptoms	19.9	9.9	35.8	276	77.9	12
Attentional Difficulties	15.9	8.7	27.3	413	77.8	13
Autism Spectrum Disorders	5.5	2.5	11.8	124	0.0	4

Behavioral Symptoms	28.4	22.2	35.4	1020	73.6	26
Anxiety and Related Symptoms	16.6	5.4	41.0	189	83.2	7
Other Mood Symptoms	24.0	8.8	50.8	114	78.5	4
Disinhibition	25.9	17.6	36.4	326	60.8	20
Eating Disorders	26.9	11.6	50.8	19	0.0	2
OCD Symptoms	19.7	3.8	60.3	50	73.8	4
Emotional Instability	10.3	5.8	17.7	133	1.8	8
Apathy	22.2	11.5	38.6	36	0.0	3

Supplementary Table 5: Meta-analytic estimate results for all cause encephalitis stratified by age group

NOS – Not otherwise stated. **OCD** - obsessive compulsive disorder

Adults only						
**Symptom**	**Prevalence (%)**	**95% Lower CI (%)**	**95% Higher CI (%)**	**Number of patients**	**I2**	**Number of cohorts**
Anxiety & Related Symptoms	60.8	42.2	76.7	259	72.3	9
Other Mood Symptoms	75.2	68.6	80.8	199	0.0	5
Disinhibition	27.6	12.8	49.9	733	90.8	9
Depressive Symptoms	32.3	22.3	44.1	762	76.6	13
OCD Symptoms	13.7	9.7	19.0	219	0.0	7
Psychotic Symptoms	25.9	8.9	55.5	652	92.0	6
Substance Use Disorders	9.8	4.5	20.2	199	20.4	5
Manic Symptoms	4.4	1.6	11.2	678	54.6	8
Personality Disorders	10.4	6.8	15.6	199	0.0	5
Impulse Control Disorders	10.2	6.6	15.3	199	0.0	5
Sexual Disorders	36.1	29.8	42.9	210	0.0	6
Emotional Instability	35.9	20.8	54.4	731	89.3	9
**Children only**						
**Symptom**	**Prevalence (%)**	**95% Lower CI (%)**	**95% Higher CI (%)**	**Number of patients**	**I2**	**Number of cohorts**
Attentional Difficulties	32.8	18.2	51.7	95	51.5	5
Autism Spectrum Disorders	8.8	2.9	24.0	36	0.0	2
Behavioral Symptoms	25.1	14.5	39.7	589	89.1	9
Disinhibition	37.8	22.4	56.1	65	24.0	4
Depressive Symptoms	15.2	3.7	45.6	60	57.3	2
Psychotic Symptoms	5.0	0.3	48.3	138	86.4	2

Supplementary Table 6: Meta-analytic estimate results for all cause infectious encephalitis stratified by age group

NOS – Not otherwise stated. **OCD** - obsessive compulsive disorder

**Adults only**						
**Symptom**	**Prevalence (%)**	**95% Lower CI (%)**	**95% Higher CI (%)**	**Number of patients**	**I2**	**Number of cohorts**
Psychotic Symptoms	13.7	7.4	24.0	442	72.1	19
Depressive Symptoms	30.8	23.9	38.7	1059	76.1	30
Anxiety and Related Symptoms	28.4	16.9	43.6	506	83.4	20
Other Mood Symptoms	62.9	19.0	92.4	177	90.7	5
Disinhibition	34.8	17.2	57.8	119	72.5	8
OCD Symptoms	13.3	5.8	27.7	58	14.1	4
Substance Use Disorders	6.3	2.2	16.8	58	0	4
Personality Disorders	16.8	8.9	29.3	58	0	4
Manic Symptoms	12.5	7.2	20.8	109	0.0	6
Impulse Control Disorders	9.7	4.5	19.6	68	0.0	6
Sexual Disorders	24.9	15.4	37.5	58	0.0	4
Emotional Instability	37.9	22.7	55.9	266	80.3	12
Behavioural Symptoms	19.9	6.6	46.6	217	85.1	6
Attentional Difficulties	27.6	8.5	61.0	54	51.2	2
Apathy	28.5	5.6	73.0	55	82.5	3
Suicidality and Self-Harm	3.7	1.0	12.7	144	20.5	4
**Children only**						
**Symptom**	**Prevalence (%)**	**95% Lower CI (%)**	**95% Higher CI (%)**	**Number of patients**	**I2**	**Number of cohorts**
Psychotic Symptoms	25.2	16.0	37.4	66	0.0	5
Depressive Symptoms	20.7	7.3	46.2	152	77.1	8
Anxiety and Related Symptoms	24.0	6.3	59.6	131	86.4	5

Other Mood Symptoms	24.0	8.8	50.8	114	78.5	4
Disinhibition	25.5	15.8	38.5	220	59.4	15
OCD Symptoms	19.7	3.8	60.3	50	73.8	4
Emotional Instability	7.6	3.5	15.6	101	0.0	6
Behavioural Symptoms	28.1	20.7	37.0	337	49.7	15
Attentional Difficulties	10.3	4.2	22.7	278	74.9	7
Apathy	22.2	11.5	38.6	36	0.0	3
Autism Spectrum Disorders	3.5	1.1	10.3	88	0.0	2

Supplementary Table 7: Meta-analytic estimate results for all cause autoimmune encephalitis stratified by age group

NOS – Not otherwise stated. **OCD** - obsessive compulsive disorder

Symptom	Adult Prevalence	Child Prevalence	Z-score	P-value
Anxiety & Related Symptoms	0.280	0.166	1.052	0.293
Apathy	0.232	0.222	0.061	0.952
Attentional Difficulties	0.146	0.159	-0.107	0.915
Behavioral Symptoms	0.204	0.284	-0.836	0.403
Depressive Symptoms	0.293	0.199	1.299	0.194
Disinhibition	0.181	0.259	-1.056	0.291
Eating Disorders	0.101	0.269	-1.653	0.098
Emotional Instability	0.263	0.103	2.383	0.017
OCD Symptoms	0.363	0.197	1.135	0.256
Other Mood Symptoms	0.403	0.240	0.683	0.495
Psychotic Symptoms	0.087	0.163	-1.414	0.157

Supplementary table 8: Symptom prevalence comparisons between adult and childhood and encephalitis (all-cause).

**Follow up time**					
**Symptom**	**Regression coefficient**	**Lower 95% CI**	**Upper 95% CI**	**P value**	**Number of cohorts**
Anxiety & Related Symptoms	0.0063	-0.01998	0.032581	0.638495	5
Disinhibition	-0.01678	-0.03297	-0.0006	0.042153	6
Depressive Symptoms	-0.00523	-0.01863	0.008172	0.444493	9
**Age**					
**Symptom**	**Regression coefficient**	**Lower 95% CI**	**Upper 95% CI**	**P value**	**Number of cohorts**
Attentional Difficulties	0.1376	-0.2063	0.4816	0.4328	5
Behavioral Symptoms	0.0082	-0.0154	0.0319	0.4957	9
Anxiety & Related Symptoms	0.0795	0.0306	0.1284	0.0014	10
Other Mood Symptoms	-0.0571	-0.5161	0.4018	0.8072	5
Disinhibition	0.0010	-0.0165	0.0185	0.9105	12
Depressive Symptoms	0.0098	-0.0125	0.0321	0.3900	17
OCD Symptoms	0.0667	-0.0341	0.1674	0.1946	8
Psychotic Symptoms	0.0209	0.0009	0.0409	0.0404	6
Substance Use Disorders	0.4190	-0.0475	0.8855	0.0784	5
Manic Symptoms	0.0717	-0.1620	0.3054	0.5476	7
Personality Disorders	0.0749	-0.4215	0.5712	0.7675	5
Impulse Control Disorders	0.1198	-0.3867	0.6262	0.6430	5
Sexual Disorders	0.0367	-0.0089	0.0824	0.1146	7
Emotional Instability	0.0294	-0.0314	0.0902	0.3437	9

Female sex					
**Symptom**	**Regression coefficient**	**Lower 95% CI**	**Upper 95% CI**	**P value**	**Number of cohorts**
Attentional Difficulties	-0.02762	-0.08988	0.034647	0.38467197	5
Behavioral Symptoms	0.005432	-0.05874	0.069601	0.86822754	9
Anxiety & Related Symptoms	0.035298	-0.02658	0.097177	0.26354676	9
Disinhibition	0.013209	-0.02836	0.054777	0.53342791	11
Depressive Symptoms	0.010627	-0.03225	0.053506	0.62715924	15

Supplementary table 9: meta regressions for symptoms prevalence and age, female sex and length of follow up for infectious encephalitis

Follow up time					
**Symptom**	**Regression coefficient**	**Lower 95% CI**	**Upper 95% CI**	**P value**	**Number of cohorts**
Psychotic Symptoms	-0.0110	-0.0346	0.0126	0.3601	11
Depressive Symptoms	-0.0415	-0.0622	-0.0207	0.0001	16
Anxiety & Related Symptoms	-0.0367	-0.0607	-0.0127	0.0027	7
Disinhibition	-0.0189	-0.0410	0.0031	0.0925	12
Emotional Instability	0.0925	0.0543	0.1308	< 0.0001	6
Behavioral Symptoms	-0.0059	-0.0232	0.0113	0.5022	9
**Age**					
**Symptom**	**Regression coefficient**	**Lower 95% CI**	**Upper 95% CI**	**P value**	**Number of cohorts**
Psychotic Symptoms	0.0114	-0.0212	0.0440	0.4914	17
Depressive Symptoms	0.0206	-0.0014	0.0426	0.0660	24

Anxiety & Related Symptoms	0.0301	-0.0129	0.0730	0.1699	15
Other Mood Symptoms	0.0578	0.0154	0.1003	0.0076	7
Disinhibition	0.0368	0.0077	0.0659	0.0132	13
OCD Symptoms	-0.0193	-0.0815	0.0429	0.5430	6
Manic Symptoms	-0.0463	-0.1181	0.0256	0.2071	5
Impulse Control Disorders	0.0116	-0.0696	0.0929	0.7790	6
Emotional Instability	0.0200	-0.0196	0.0597	0.3226	9
Behavioral Symptoms	-0.0210	-0.0487	0.0067	0.1380	11
**Female sex**					
**Symptom**	**Regression coefficient**	**Lower 95% CI**	**Upper 95% CI**	**P value**	**Number of cohorts**
Psychotic Symptoms	0.0084	-0.0237	0.0406	0.6070	17
Depressive Symptoms	-0.0147	-0.0318	0.0024	0.0928	28
Anxiety & Related Symptoms	0.02237	-0.0147	0.0594	0.2369	16
Other Mood Symptoms	-0.0191	-0.1231	0.0850	0.7194	6
Disinhibition	0.0199	-0.0108	0.0506	0.2031	14
Emotional Instability	0.0177	-0.0140	0.0494	0.2732	13
Behavioral Symptoms	0.0011	-0.0190	0.0212	0.9113	25
Attentional Difficulties	-0.0217	-0.0885	0.0450	0.5234	8
Apathy	-0.0479	-0.0719	-0.0238	0.0001	6
Suicidality and Self-Harm	0.0069	-0.0885	0.1024	0.8868	5

Supplementary table 10: meta regressions for symptoms prevalence and age, female sex and length of follow up for autoimmune encephalitis

Symptom	Autoimmune Prevalence	Infectious Prevalence	Z-score	P-value
Other Mood Symptoms	0.309	0.752	-3.398	0.001
Manic Symptoms	0.115	0.044	1.853	0.064
Sexual Disorders	0.249	0.354	-1.613	0.107
Personality Disorders	0.168	0.104	1.126	0.260
Autism Spectrum Disorders	0.035	0.088	-0.897	0.370
Attentional Difficulties	0.128	0.218	-0.877	0.380
Substance Use Disorders	0.063	0.098	-0.642	0.521
OCD Symptoms	0.170	0.131	0.504	0.614
Behavioral Symptoms	0.278	0.251	0.376	0.707
Psychotic Symptoms	0.156	0.183	-0.285	0.775
Disinhibition	0.269	0.249	0.253	0.800
Emotional Instability	0.299	0.319	-0.207	0.836
Depressive Symptoms	0.296	0.284	0.207	0.836
Anxiety & Related Symptoms	0.288	0.314	-0.206	0.836
Impulse Control Disorders	0.097	0.102	-0.102	0.919

Supplementary table 11: Symptom prevalence comparisons between autoimmune and infective encephalitis.

**Herpes Simplex Virus**						
**Symptom**	**Prevalence (%)**	**95% Lower CI (%)**	**95% Higher CI (%)**	**Number of patients**	**I^2**	**Number of cohorts**
Behavioral Symptoms NOS	29.2%	14.4%	50.2%	253	80.9	3
Anxiety and Related Symptoms	40.9%	8.8%	83.3%	241	95.3	4
Disinhibited Behavior	39.1%	17.5%	66.1%	212	83.7	3
Depressive Symptoms	29.8%	18.3%	44.7%	296	75.8	6
OCD Symptoms	11.9%	6.5%	20.8%	207	17.8	3
Sexual Disorders	24.7%	6.0%	62.8%	178	64.3	2
Emotional Instability	48.5%	35.5%	61.7%	178	23.8	2
Eating disorder	5.6%	1.4%	19.8%	40	0.0	2
**Epstein-Barr Virus**						
**Symptom**	**Prevalence (%)**	**95% Lower CI (%)**	**95% Higher CI (%)**	**Number of patients**	**I2**	**Number of cohorts**
Disinhibited Behavior	17.4%	4.2%	50.2%	13	0.0	2
Sexual Disorders	17.4%	4.2%	50.2%	13	0.0	2
**Japanese Encephalitis**						
**Symptom**	**Prevalence (%)**	**95% Lower CI (%)**	**95% Higher CI (%)**	**Number of patients**	**I2**	**Number of cohorts**
Behavioral Symptoms	14.6%	4.2%	40.0%	105	78.0	2
Disinhibition	10.5%	0.3%	82.3%	455	85.4	2
Depressive Symptoms	30.8%	7.9%	69.8%	462	76.3	3
Psychotic Symptoms	8.5%	0.3%	74.9%	551	91.5	3

Manic Symptoms	3.3%	0.3%	29.9%	455	64.8	2
Emotional Instability	24.1%	6.7%	58.2%	462	55.3	3
**Tick Borne encephalitis**						
**Symptom**	**Prevalence (%)**	**95% Lower CI (%)**	**95% Higher CI (%)**	**Number of patients**	**I2**	**Number of cohorts**
Behavioral Symptoms NOS	23.8%	13.3%	38.9%	50	0.0	2
Disinhibition	38.1%	27.5%	49.9%	119	27.4	4
Depressive Symptoms	21.3%	12.8%	33.5%	67	0.0	3
Psychotic Symptoms	22.7%	9.5%	45.1%	50	41.6	2
Manic Symptoms	8.5%	2.1%	28.5%	25	0.0	2
Emotional Instability	21.4%	6.6%	51.2%	76	66.5	3
**Varicella Zoster Virus**						
**Symptom**	**Prevalence (%)**	**95% Lower CI (%)**	**95% Higher CI (%)**	**Number of patients**	**I2**	**Number of cohorts**
Anxiety and Related Symptoms	33.6%	0.4%	98.6%	33	90.3	2
Disinhibited Behavior	21.6%	4.1%	63.8%	32	65.4	2
Depressive Symptoms	23.1%	3.6%	70.8%	32	72.3	2
**West Nile Virus**						
**Symptom**	**Prevalence (%)**	**95% Lower CI (%)**	**95% Higher CI (%)**	**Number of patients**	**I2**	**Number of cohorts**
Depressive Symptoms	54.5%	17.7%	86.9%	58	87.5	2
**Nipah Virus**						
**Symptom**	**Prevalence (%)**	**95% Lower CI (%)**	**95% Higher CI (%)**	**Number of patients**	**I2**	**Number of cohorts**
Behavioral Symptoms	31.2%	16.4%	51.2%	26	0.0	2

NOS						
Depressive Symptoms	57.7%	38.5%	74.8%	26	0.0	2

Supplementary table 12: Infectious encephalitis results stratified by agent

Herpes Simplex Virus (adults)						
**Symptom**	**Prevalence (%)**	**95% Lower CI (%)**	**95% Higher CI (%)**	**Number of patients**	**I^2**	**Number of cohorts**
Anxiety & Related Symptoms	62.9%	32.3%	85.8%	208	89.2	3
Disinhibition	48.3%	33.4%	63.5%	178	32.8	2
Depressive Symptoms	41.3%	35.1%	47.8%	234	0.0	4
OCD Symptoms	14.0%	9.6%	20.0%	178	0.0	2
Sexual Disorders	24.7%	6.0%	62.8%	178	64.3	2
Emotional Instability	48.5%	35.5%	61.7%	178	23.8	2
**Herpes Simplex Virus (children)**						
Behavioral Symptoms	18.9%	14.1%	25.0%	224	2.4	2
**Japanese Encephalitis (adults)**						
Disinhibition	10.5%	0.3%	82.3%	455	85.4	2
Depressive Symptoms	30.8%	7.9%	69.8%	462	76.3	3
Psychotic Symptoms	23.5%	0.2%	97.9%	455	90.6	2
Manic Symptoms	3.3%	0.3%	29.9%	455	64.8	2
Emotional Instability	24.1%	6.7%	58.2%	462	55.3	3
**Japanese Encephalitis (children)**						
Behavioral Symptoms	14.6%	4.2%	40.0%	105	78.0	2
**Tick borne encephalitis (adults)**						
Disinhibition	30.5%	20.8%	42.3%	69	0.0	2

Depressive Symptoms	14.0%	3.1%	45.2%	25	38.3	2
Manic Symptoms	8.5%	2.1%	28.5%	25	0.0	2
Emotional Instability	24.9%	4.3%	70.9%	69	84.2	2
**Tick borne encephalitis (children)**						
Disinhibition	46.0%	32.8%	59.8%	50	0.0	2
Behavioral Symptoms	23.8%	13.3%	38.9%	50	0.0	2

Supplementary table 13: Infectious encephalitis results stratified by agent and age group

**Anti-NMDAR**						
**Symptom**	**Prevalence (%)**	**95% Lower CI (%)**	**95% Higher CI (%)**	**Number of patients**	**I²**	**Number of cohorts**
Psychotic Symptoms	15.8%	8.5%	27.5%	249	64.6	12
Depressive Symptoms	18.7%	11.6%	28.7%	456	72.2	21
Anxiety and Related Symptoms	25.0%	11.0%	47.4%	340	88.0	13
Other Mood Symptoms	20.8%	3.8%	63.7%	162	90.9	5
Disinhibition	23.2%	14.9	34.4	305	61.9	17
OCD Symptoms	8.3%	3.7%	17.3%	78	0.0	4
Manic Symptoms	11.0%	5.3%	21.4%	66	0.0	2
Impulse Control Disorders	8.0%	2.8%	20.5%	43	0.0	2
Emotional Instability	30.2%	15.8%	50.0%	266	83.4	12
Behavioral Symptoms	35.0%	26.4%	44.6%	297	48.8	16
Attentional Difficulties	10.8%	4.4%	24.2%	260	73.9	7
Apathy	17.6%	9.8%	29.6%	87	0.0	5
Autism Spectrum Disorders	3.5%	1.1%	10.3%	88	0.0	2
Suicidality and Self-harm	7.2%	1.4%	28.9%	20	0.0	2
**Anti-LGI 1**						
**Symptom**	**Prevalence (%)**	**95% Lower CI (%)**	**95% Higher CI (%)**	**Number of patients**	**I²**	**Number of cohorts**
Psychotic Symptoms	16.1%	0.2%	94.6%	44	88.9	2

Depressive Symptoms	28.8%	19.2%	40.6%	132	39.3	5
Anxiety and Related Symptoms	36.8%	7.9%	79.7%	112	89.8	4
Manic Symptoms	2.9%	0.4%	18.0%	33	0.0	2
Emotional instability	24.0%	6.5	58.9	151	90.0	4
**Anti-GAD**						
**Symptom**	**Prevalence (%)**	**95% Lower CI (%)**	**95% Higher CI (%)**	**Number of patients**	**I^2**	**Number of cohorts**
Psychotic Symptoms	18.3%	5.7%	45.7%	30	43.4	2
Depressive Symptoms	52.8%	25.1%	79.0%	30	56.4	2
Anxiety and Related Symptoms	44.7%	3.7%	94.5%	30	89.6	2
**Anti-GABAR**						
**Symptom**	**Prevalence (%)**	**95% Lower CI (%)**	**95% Higher CI (%)**	**Number of patients**	**I^2**	**Number of cohorts**
Psychotic Symptoms	18.5%	9.1%	34.1%	38	0.0	2
Depressive Symptoms	51.2%	35.3%	66.8%	37	0.0	2
**Anti-CASPR2**						
**Symptom**	**Prevalence (%)**	**95% Lower CI (%)**	**95% Higher CI (%)**	**Number of patients**	**I^2**	**Number of cohorts**
Depressive Symptoms	58.4%	42.1%	73.2%	36	0.0	2

Supplementary table 14: Autoimmune encephalitis results stratified by agent

	All NMDA studies	CSF-confirmed studies		Non-CSF-confirmed studies		Meta-regression	
Symptom	Prevalence (95% CI)	K	Prevalence (95% CI)	K	Prevalence (95% CI)	β (95% CI)	P-value
Behavioral Symptoms	0.356 (0.273–0.448)	4	0.571 (0.405–0.723)	12	0.319 (0.233–0.42)	1.02 (0.155–1.89)	0.021
Psychotic Symptoms	0.158 (0.0848–0.275)	6	0.0841 (0.0299–0.215)	6	0.26 (0.137–0.439)	-1.21 (-2.54–0.111)	0.073
Other Mood Symptoms	0.208 (0.0376–0.637)	2	0.0456 (0.00621–0.268)	3	0.403 (0.0666–0.865)	-2.76 (-6.38–0.862)	0.135
Disinhibition	0.232 (0.149–0.344)	6	0.143 (0.0525–0.335)	12	0.276 (0.172–0.412)	-0.806 (-2.02–0.405)	0.192
Emotional Instability	0.302 (0.158–0.5)	3	0.147 (0.0295–0.496)	9	0.37 (0.197–0.585)	-1.23 (-3.1–0.647)	0.199
Apathy	0.176 (0.0983–0.296)	2	0.0618 (0.00376–0.535)	3	0.203 (0.109–0.347)	-1.14 (-3.01–0.735)	0.234
Depressive Symptoms	0.187 (0.116–0.287)	10	0.243 (0.14–0.386)	11	0.138 (0.0618–0.281)	0.595 (-0.536–1.72)	0.302
OCD Symptoms	0.0826 (0.0374–0.173)	2	0.122 (0.0302–0.382)	2	0.0686 (0.026–0.169)	0.633 (-1.17–2.44)	0.492
Attentional Difficulties	0.108 (0.044–0.242)	3	0.0802 (0.045–0.139)	4	0.131 (0.0304–0.422)	-0.654 (-2.68–1.37)	0.527
Anxiety & Related Symptoms	0.25 (0.11–0.474)	5	0.205 (0.0322–0.667)	8	0.267 (0.101–0.542)	-0.26 (-2.43–1.91)	0.814

Supplementary table 15: NMDA receptor encephalitis results stratified by CSF confirmation status

**Anti-NMDAR (adults)**						
**Symptom**	**Prevalence (%)**	**95% Lower CI (%)**	**95% Higher CI (%)**	**Number of patients**	**I^2**	**Number of cohorts**
Anxiety & Related Symptoms	27.3%	7.4%	63.6%	132	86.2	6
Disinhibition	35.9%	11.7%	70.3%	58	73.2	3
Depressive Symptoms	15.9%	8.4%	28.2%	191	58.1	10
Psychotic Symptoms	14.5%	5.0%	35.2%	110	66.7	6
Impulse Control Disorders	8.0%	2.8%	20.5%	43	0.0	2
Emotional Instability	51.4%	30.6%	71.7%	70	54.8	5
Behavioral Symptoms	21.8%	6.3%	53.8%	12	0.0	2
Suicidality and Self-Harm	7.2%	1.4%	28.9%	20	0.0	2
**Anti-NMDAR (children)**						
**Symptom**	**Prevalence (%)**	**95% Lower CI (%)**	**95% Higher CI (%)**	**Number of patients**	**I^2**	**Number of cohorts**
Psychotic Symptoms	22.4%	6.5%	54.4%	19	28.8	2
Depressive Symptoms	15.7%	5.5%	37.3%	142	70.1	7
Anxiety and Related Symptoms	15.1%	4.5%	40.0%	121	78.4	4
Other Mood Symptoms	15.2%	6.2%	32.8%	87	43.3	3
Disinhibition	22.7%	14.7%	33.2%	210	43.5	14
OCD Symptoms	8.7%	2.8%	23.9%	40	0.0	3

Emotional Instability	6.4%	2.8%	14.1%	96	0.0	5
Attentional Difficulties	10.7%	3.9%	26.2%	251	78.7	6
Apathy	22.2%	11.5%	38.6%	36	0.0	3
Autism Spectrum Disorders	3.5%	1.1%	10.3%	88	0.0	2
**Anti GABA-R (adults)**						
**Symptom**	**Prevalence (%)**	**95% Lower CI (%)**	**95% Higher CI (%)**	**Number of patients**	**I^2**	**Number of cohorts**
Psychotic Symptoms	18.5%	9.1%	34.1%	38	0.0	2
Depressive Symptoms	51.2%	35.3%	66.8%	37	0.0	2
**Anti-LGI 1 (adults)**						
**Symptom**	**Prevalence (%)**	**95% Lower CI (%)**	**95% Higher CI (%)**	**Number of patients**	**I^2**	**Number of cohorts**
Psychotic Symptoms	16.1%	0.2%	94.6%	44	88.9	2
Depressive Symptoms	28.8%	19.2%	40.6%	132	39.3	5
Anxiety and Related Symptoms	36.8%	7.9%	79.7%	112	89.8	4
Manic Symptoms	2.9%	0.4%	18.0%	33	0.0	2
Emotional instability	24.0%	6.5	58.9	151	90.0	4

Supplementary table 16: Autoimmune encephalitis results stratified by agent and age group

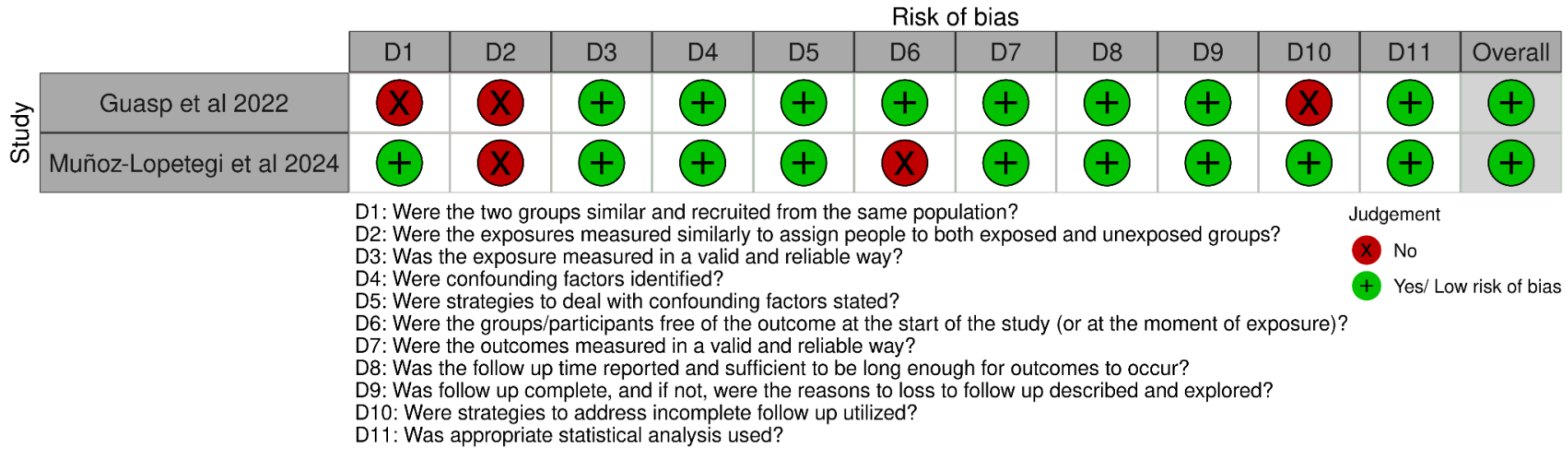

Supplementary Figure 1: Risk of bias summary for included cohort studies assessing psychiatric and behavioral outcomes following encephalitis

Risk of bias

Study	D1	D2	D3	D4	D5	D6	D7	D8	D9	D10	Overall
Wang 2019	+	+	+	+	+	X	+	+	+	+	+
Sola-Valls et al 2020	+	+	+	+	+	X	X	+	+	+	+
McKeon et al 2016	+	+	+	+	+	X	X	+	+	+	+
Cai et al 2020	+	+	+	X	+	+	+	+	+	+	+
Caparros-Lefebvre et al 1996	+	+	+	X	X	+	X	+	+	+	-
Engman et al 2012	+	+	+	+	+	+	X	X	+	+	+
Harris et al 2020	+	X	+	+	+	+	X	+	+	+	+
Lin et al 2022	+	+	+	X	X	+	+	+	+	+	+
Griska et al 2024	+	+	X	+	X	+	+	+	+	+	+
Chen et al 2021	+	+	X	+	X	+	+	+	+	+	+

D1: Were the groups comparable other than the presence of disease in cases or the absence of disease in controls?
D2: Were cases and controls matched appropriately?
D3: Were the same criteria used for identification of cases and controls?
D4: Was exposure measured in a standard, valid and reliable way?
D5: Was exposure measured in the same way for cases and controls?
D6: Were confounding factors identified?
D7: Were strategies to deal with confounding factors stated?
D8: Were outcomes assessed in a standard, valid and reliable way for cases and controls?
D9: Was the exposure period of interest long enough to be meaningful?
D10: Was appropriate statistical analysis used?

Judgement
X No
\- Moderate risk of bias
\+ Yes/ Low risk of bias

Supplementary Figure 2: Risk of bias summary for included case control studies assessing psychiatric and behavioral outcomes following encephalitis

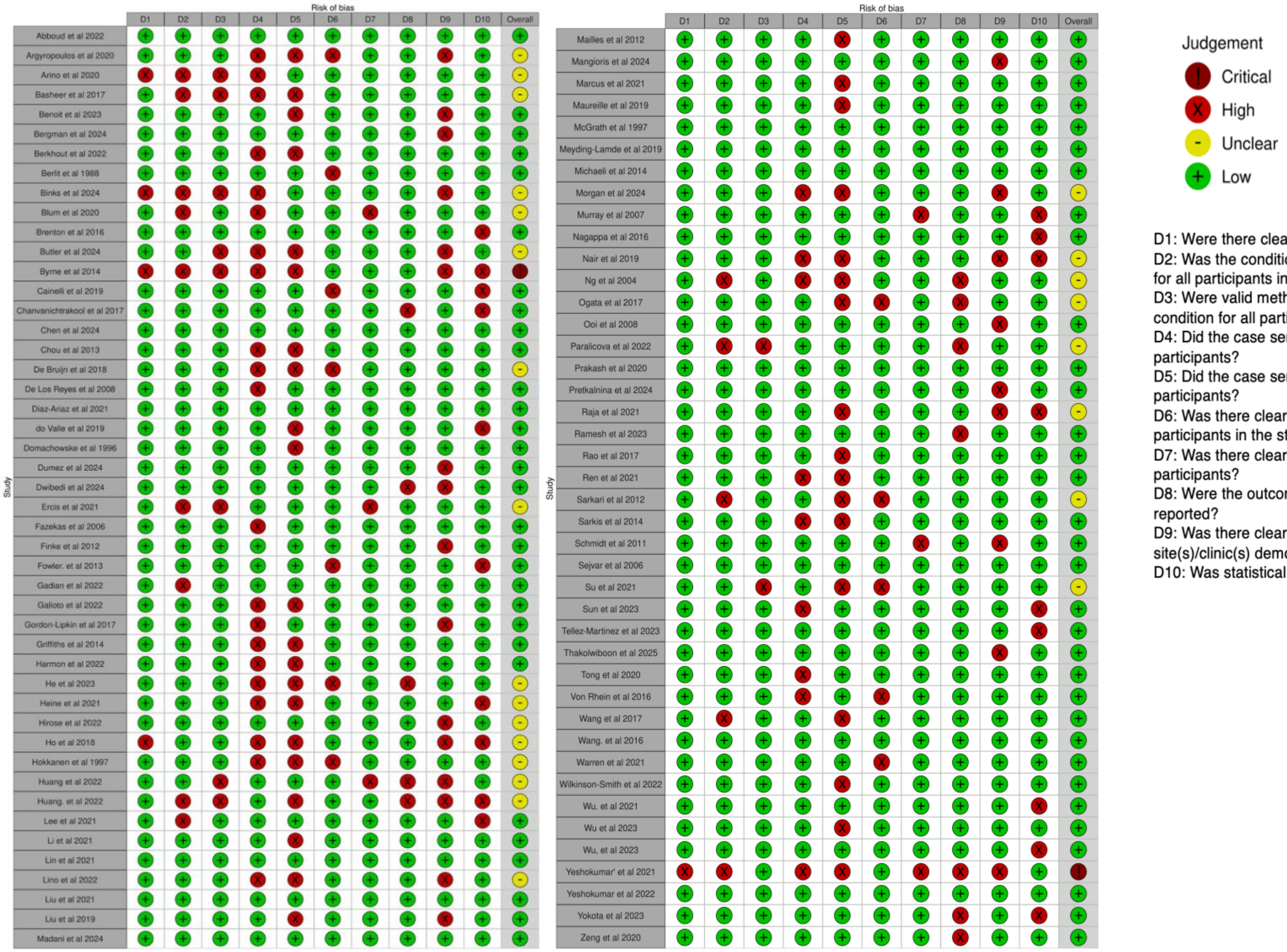

D1: Were there clear criteria for inclusion in the case series?
D2: Was the condition measured in a standard, reliable way for all participants included in the case series?
D3: Were valid methods used for identification of the condition for all participants included in the case series?
D4: Did the case series have consecutive inclusion of participants?
D5: Did the case series have complete inclusion of participants?
D6: Was there clear reporting of the demographics of the participants in the study?
D7: Was there clear reporting of clinical information of the participants?
D8: Were the outcomes or follow up results of cases clearly reported?
D9: Was there clear reporting of the presenting site(s)/clinic(s) demographic information?
D10: Was statistical analysis appropriate?

Supplementary Figure 3: Risk of bias summary for included case series assessing psychiatric and behavioral outcomes following encephalitis

Supplementary Methods 1: database search strategy

1. encephalitis*.mp. or *encephalitis/
2. (mental health or mental disorder* or psychiatric or psychiatry or neuropsychiatric or neuropsychiatry or psychological or psychology or neuropsychological or neuropsychology or Behavior* or neuroBehavior*or behavior* or neurobehavior* or mental wellbeing or wellbeing or well-being or depress* or psychosis* or anxiety or emotion* or hypersensitivit* or psychiatric disorder* or panic disorder or obsessive- compulsive disorder* or eating disorder* or personality disorder* or post-traumatic stress disorder*or psychotic disorder* or schizophrenia).ti,ab,kw
3. (outcome* or sequel* or consequence* or following* or change or aspects*).mp.
4. 1 and 2 and 3
5. Limit 4 to (human and English language)

Supplementary reference list

1. Abboud H, Briggs F, Buerki R, et al: Residual symptoms and long-term outcomes after all-cause autoimmune encephalitis in adults. J. Neurol. Sci. 2022; 434: 120124.
2. Berkhout A, Kapoor V, Heney C, et al: Epidemiology and long-term neurological sequelae of childhood herpes simplex CNS infection. J. Paediatr. Child Health 2022; 58: 1372–1378.
3. Butler M, Abdat Y, Zandi M, et al: Mental health outcomes of encephalitis: An international web-based study. Eur. J. Neurol. 2024; 31: e16083.
4. Diaz-Arias LA, Yeshokumar AK, Glassberg B, et al: Fatigue in Survivors of Autoimmune Encephalitis. Neurol. Neuroimmunol. NeuroInflammation 2021; 8: e1064.
5. Ercis M, Dogan FU, Berberoglu E, et al: Neuropsychiatric outcomes of patients with encephalitis: A case series with long-term follow-up. Dusunen Adam J. Psychiatry Neurol. Sci. 2023; 36: 50.
6. Gadian J, Eyre M, Konstantoulaki E, et al: Neurological and cognitive outcomes after antibody-negative autoimmune encephalitis in children. Dev. Med. Child Neurol. 2022; 64: 649–653.
7. Guasp M, Rosa-Justicia M, Muñoz-Lopetegi A, et al: Clinical characterisation of patients in the post-acute stage of anti-NMDA receptor encephalitis: a prospective cohort study and comparison with patients with schizophrenia spectrum disorders. Lancet Neurol. 2022; 21: 899–910.
8. Harmon A, Stingl C, Rikhi A, et al: Pediatric GAD-65 Autoimmune Encephalitis: Assessing Clinical Characteristics and Response to Therapy With a Novel Assessment Scale. Pediatr. Neurol. 2022; 128: 25–32.
9. He L, Zhang X and Wu J: Ovarian teratoma-associated anti-N-methyl-D-aspartate receptor encephalitis: a clinical analysis of 5 cases. Ginekol. Pol. 2023; 94: 451–455.
10. Hirose S, Hara M, Kamei S, et al: Characteristics of clinical relapses and patient-oriented long-term outcomes of patients with anti-N-methyl-d-aspartate receptor encephalitis. J. Neurol. 2022; 269: 2486–2492.
11. Huang A, Wang J, Feng D, et al: Case Analysis and Literature Review of Thirteen Patients with Autoimmune Encephalitis. Dis. Markers 2022; 2022: 4802480.
12. Huang X, Fan C, Gao L, et al: Clinical Features, Immunotherapy, and Outcomes of Anti-Leucine-Rich Glioma-Inactivated-1 Encephalitis. J. Neuropsychiatry Clin. Neurosci. 2022; 34: 141–148.
13. Lee S, Kim HD, Lee JS, et al: Clinical Features and Treatment Outcomes of Seronegative Pediatric Autoimmune Encephalitis. J. Clin. Neurol. 2021; 17: 300–306.
14. Li X, Hou C, Wu W-L, et al: Pediatric anti-N-methyl-d-aspartate receptor encephalitis in southern China: Analysis of 111 cases. J. Neuroimmunol. 2021; 352: 577479.
15. Lin H-Y, Chen Y-L, Chou P-H, et al: Long-term psychiatric outcomes in youth with enterovirus A71 central nervous system involvement. Brain Behav. Immun. - Health 2022; 23: 100479.
16. Lin J, Li C, Li A, et al: Long-term cognitive and neuropsychiatric outcomes of anti-GABABR encephalitis patients: A prospective study. J. Neuroimmunol. 2021; 351: 577471.
17. Lino A, Erickson TA, Nolan MS, et al: A Preliminary Study of Proinflammatory Cytokines and Depression Following West Nile Virus Infection. Pathogens 2022; 11: 650.
18. Marcus L and Ness JM: Pediatric N-Methyl-d-Aspartate (NMDA) Receptor Encephalitis, With and Without Herpes Encephalitis. J. Child Neurol. 2021; 36: 743–751.
19. Paraličová Z, Sekula J, Jarčuška P, et al: Outbreak of Alimentary Tick-Borne Encephalitis in Eastern Slovakia: An Analysis of Affected Patients and Long-Term Outcomes. Pathogens 2022; 11: 433.
20. Ramesh R, Hazeena P, Shanmugam S, et al: Autoimmune encephalitis: an observational study from South India. Egypt. J. Neurol. Psychiatry Neurosurg. 2023; 59: 25.
21. Ren C, Zhang W, Ren X, et al: Clinical Features and Outcomes of Anti-N-Methyl-d-Aspartate Receptor Encephalitis in Infants and Toddlers. Pediatr. Neurol. 2021; 119: 27–33.
22. Su Q, Xie Z-X, He F, et al: Adults with severe Japanese encephalitis: a retrospective analysis of 9 cases in Linyi, China. Neurol. Sci. 2021; 42: 2811–2817.
23. Sun Y, Qin X, Huang D, et al: Anti-amphiphysin encephalitis: Expanding the clinical spectrum. Front. Immunol. 2023; 14. Available at: https://www.frontiersin.org/journals/immunology/articles/10.3389/fimmu.2023.1084883/full, accessed July 7, 2025.
24. Tellez-Martinez A, Restrepo-Martinez M, Espinola-Nadurille M, et al: Suicidal Thoughts and Behaviors in Anti-NMDA Receptor Encephalitis: Psychopathological Features and Clinical Outcomes. J. Neuropsychiatry Clin. Neurosci. 2023; 35: 368–373.
25. Warren N, O’Gorman C, McKeon G, et al: Psychiatric management of anti-NMDAR encephalitis: a cohort analysis. Psychol. Med. 2021; 51: 435–440.

26. Wilkinson-Smith A, Blackwell LS and Howarth RA: Neuropsychological outcomes in children and adolescents following anti-NMDA receptor encephalitis. Child Neuropsychol. 2022; 28: 212–223.
27. Wu H, Wu C, Zhou Y, et al: Catatonia in adult anti-NMDAR encephalitis: an observational cohort study. BMC Psychiatry 2023; 23: 94.
28. Wu P-M, Teng C-K, Chou Y-Y, et al: Precocious puberty as a consequence of anti-NMDA receptor encephalitis in children. Pediatr. Neonatol. 2021; 62: 361–368.
29. Wu P-Y, Chi C-S, Tsai C-R, et al: Long-Term Outcome of Pediatric Patients with Anti-NMDA Receptor Encephalitis in a Single Center. Child. Basel Switz. 2023; 10: 182.
30. Yokota Y, Hirose S, Hara M, et al: Long-term outcomes and health-related quality of life in patients with autoimmune encephalitis: An observational study. Medicine (Baltimore) 2023; 102: e35162.
31. Zeng W, Cao L, Zheng J, et al: Clinical characteristics and long-term follow-up of seven cases of anti-GABABR encephalitis in patients of Han Chinese descent. Neurol. Sci. Off. J. Ital. Neurol. Soc. Ital. Soc. Clin. Neurophysiol. 2020; 41: 373–378.
32. Wang K, Chen Z, Wu D, et al: Early second-line therapy is associated with improved episodic memory in anti-NMDA receptor encephalitis. Ann. Clin. Transl. Neurol. 2019; 6: 1202–1213.
33. Tong L-L, Yang X-F, Zhang S-Q, et al: Clinical and EEG characteristics analysis of autoimmune encephalitis in children with positive and negative anti-N-methyl- D-aspartate receptor antibodies. Ann. Palliat. Med. 2020; 9: 2575–2585.
34. Sejvar JJ, Hossain J, Saha SK, et al: Long-term neurological and functional outcome in Nipah virus infection. Ann. Neurol. 2007; 62: 235–242.
35. Prakash Gangwar S, Thangaraj JWV, Zaman K, et al: Sequelae Following Acute Encephalitis Syndrome Caused by Orientia Tsutsugamushi. Pediatr. Infect. Dis. J. 2020; 39: e52.
36. Ooi MH, Lewthwaite P, Lai BF, et al: The epidemiology, clinical features, and long-term prognosis of Japanese encephalitis in central sarawak, malaysia, 1997-2005. Clin. Infect. Dis. Off. Publ. Infect. Dis. Soc. Am. 2008; 47: 458–468.
37. von Rhein B, Wagner J, Widman G, et al: Suspected antibody negative autoimmune limbic encephalitis: outcome of immunotherapy. Acta Neurol. Scand. 2017; 135: 134–141.
38. Sola-Valls N, Ariño H, Escudero D, et al: Telemedicine assessment of long-term cognitive and functional status in anti-leucine-rich, glioma-inactivated 1 encephalitis. Neurol. Neuroimmunol. Neuroinflammation 2020; 7: e652.
39. Schmidt A, Bühler R, Mühlemann K, et al: Long-term outcome of acute encephalitis of unknown aetiology in adults. Clin. Microbiol. Infect. Off. Publ. Eur. Soc. Clin. Microbiol. Infect. Dis. 2011; 17: 621–626.
40. Sarkis RA, Nehme R and Chemali ZN: Neuropsychiatric and seizure outcomes in nonparaneoplastic autoimmune limbic encephalitis. Epilepsy Behav. EB 2014; 39: 21–25.
41. Sarkari NBS, Thacker AK, Barthwal SP, et al: Japanese encephalitis (JE) part II: 14 Years' follow-up of survivors. J. Neurol. 2012; 259: 58–69.
42. Rao S, Elkon B, Flett KB, et al: Long-Term Outcomes and Risk Factors Associated With Acute Encephalitis in Children. J. Pediatr. Infect. Dis. Soc. 2017; 6: 20–27.
43. Ng B-Y, Lim CCT, Yeoh A, et al: Neuropsychiatric sequelae of Nipah virus encephalitis. J. Neuropsychiatry Clin. Neurosci. 2004; 16: 500–504.
44. Nair A V, Menon J and Kuzhikkathukandiyil P: Clinical Profile and Neuropsychiatric Outcome in Children with Anti-NMDAR Encephalitis. Indian Pediatr. 2019; 56: 247–249.
45. Nagappa M, Bindu PS, Mahadevan A, et al: Clinical Features, Therapeutic Response, and Follow-Up in Pediatric Anti-N-Methyl-D-Aspartate Receptor Encephalitis: Experience from a Tertiary Care University Hospital in India. Neuropediatrics 2016; 47: 24–32.
46. Murray KO, Resnick M and Miller V: Depression after Infection with West Nile Virus1. Emerg. Infect. Dis. 2007; 13: 479–481.
47. McKeon GL, Scott JG, Spooner DM, et al: Cognitive and Social Functioning Deficits after Anti-N-Methyl-D-Aspartate Receptor Encephalitis: An Exploratory Case Series. J. Int. Neuropsychol. Soc. JINS 2016; 22: 828–838.
48. McGrath N, Anderson NE, Croxson MC, et al: Herpes simplex encephalitis treated with acyclovir: diagnosis and long term outcome. J. Neurol. Neurosurg. Psychiatry 1997; 63: 321–326.
49. Maureille A, Fenouil T, Joubert B, et al: Isolated seizures are a common early feature of paraneoplastic anti-GABAB receptor encephalitis. J. Neurol. 2019; 266: 195–206.
50. Mailles A, De Broucker T, Costanzo P, et al: Long-term Outcome of Patients Presenting With Acute Infectious Encephalitis of Various Causes in France. Clin. Infect. Dis. 2012; 54: 1455–1464.

51. Berlit P: The prognosis and long-term course of viral encephalitis. J. Neuroimmunol. 1988; 20: 117–125.
52. Blum RA, Tomlinson AR, Jetté N, et al: Assessment of long-term psychosocial outcomes in anti-NMDA receptor encephalitis. Epilepsy Behav. EB 2020; 108: 107088.
53. Cainelli E, Nosadini M, Sartori S, et al: Neuropsychological And Psychopathological Profile Of Anti-Nmdar Encephalitis: A Possible Pathophysiological Model For Pediatric Neuropsychiatric Disorders. Arch. Clin. Neuropsychol. Off. J. Natl. Acad. Neuropsychol. 2019; 34: 1309–1319.
54. Caparros-Lefebvre D, Girard-Buttaz I, Reboul S, et al: Cognitive and psychiatric impairment in herpes simplex virus encephalitis suggest involvement of the amygdalo-frontal pathways. J. Neurol. 1996; 243: 248–256.
55. de Bruijn MAAM, Aarsen FK, van Oosterhout MP, et al: Long-term neuropsychological outcome following pediatric anti-NMDAR encephalitis. Neurology 2018; 90: e1997–e2005.
56. Domachowske JB, Cunningham CK, Cummings DL, et al: Acute manifestations and neurologic sequelae of Epstein-Barr virus encephalitis in children. Pediatr. Infect. Dis. J. 1996; 15: 871–875.
57. Engman M-L, Lindström K, Sallamba M, et al: One-year Follow-up of Tick-borne Central Nervous System Infections in Childhood. Pediatr. Infect. Dis. J. 2012; 31: 570.
58. Fazekas C, Enzinger C, Wallner M, et al: Depressive symptoms following herpes simplex encephalitis--an underestimated phenomenon? Gen. Hosp. Psychiatry 2006; 28: 403–407.
59. Gordon-Lipkin E, Yeshokumar AK, Saylor D, et al: Comparative Outcomes in Children and Adults With Anti-N-Methyl-D-Aspartate (anti-NMDA) Receptor Encephalitis. J. Child Neurol. 2017; 32: 930–935.
60. Fowler Å, Forsman L, Eriksson M, et al: Tick-borne encephalitis carries a high risk of incomplete recovery in children. J. Pediatr. 2013; 163: 555–560.
61. Griffiths MJ, Lemon JV, Rayamajhi A, et al: The Functional, Social and Economic Impact of Acute Encephalitis Syndrome in Nepal – a Longitudinal Follow-Up Study. PLoS Negl. Trop. Dis. 2013; 7: e2383.
62. Hokkanen L and Launes J: Cognitive recovery instead of decline after acute encephalitis: a prospective follow up study. J. Neurol. Neurosurg. Psychiatry 1997; 63: 222–227.
63. Ho AC-C, Chan SH-S, Chan E, et al: Anti-N-methyl-d-aspartate receptor encephalitis in children: Incidence and experience in Hong Kong. Brain Dev. 2018; 40: 473–479.
64. Binks SNM, Veldsman M, Handel AE, et al: Fatigue predicts quality of life after leucine-rich glioma-inactivated 1-antibody encephalitis. Ann. Clin. Transl. Neurol. 2024; **11**: 1053–1058.
65. Harris L, Griem J, Gummery A, et al: Neuropsychological and psychiatric outcomes in encephalitis: A multi-centre case-control study. PloS One 2020; 15: e0230436.
66. de los Reyes EC, McJunkin JE, Glauser TA, et al: Periodic lateralized epileptiform discharges in La Crosse encephalitis, a worrisome subgroup: clinical presentation, electroencephalogram (EEG) patterns, and long-term neurologic outcome. J. Child Neurol. 2008; 23: 167–172.
67. Michaeli O, Kassis I, Shachor-Meyouhas Y, et al: Long-term motor and cognitive outcome of acute encephalitis. Pediatrics 2014; 133: e546-552.
68. Ogata M, Oshima K, Ikebe T, et al: Clinical characteristics and outcome of human herpesvirus-6 encephalitis after allogeneic hematopoietic stem cell transplantation. Bone Marrow Transplant. 2017; 52: 1563–1570.
69. Ariño H, Muñoz-Lopetegi A, Martinez-Hernandez E, et al: Sleep disorders in anti-NMDAR encephalitis. Neurology 2020; 95: e671–e684.
70. Basheer S, Nagappa M, Mahadevan A, et al: Neuropsychiatric Manifestations of Pediatric NMDA Receptor Autoimmune Encephalitis: A Case Series From a Tertiary Care Center in India. Prim. Care Companion CNS Disord. 2017; 19: 17m02110.
71. Brenton JN, Kim J and Schwartz RH: Approach to the Management of Pediatric-Onset Anti-N-Methyl-d-Aspartate (Anti-NMDA) Receptor Encephalitis: A Case Series. J. Child Neurol. 2016; 31: 1150–1155.
72. Chanvanichtrakool M, Likasitwattanakul S, Rongnoparat K, et al: Anti-nmda receptor encephalitis: CASE series and long-term outcomes. Southeast Asian J Trop Med Public Health 2017; 48: 232–230.
73. Chou I-J, Wang H-S, Lin J-J, et al: Limbic encephalitis in Taiwanese children and adolescence: a single center study. Pediatr. Neonatol. 2013; 54: 246–253.
74. Pretkalnina D, Grinvalde S and Kalnina E: Pediatric Autoimmune Encephalitis: A Nationwide Study in Latvia. Neuropediatrics 2024; 55: 321–326.
75. Chen L-W, Olivé-Cirera G, Fonseca EG, et al: Very Long-Term Functional Outcomes and Dependency in Children With Anti-NMDA Receptor Encephalitis. Neurol. Neuroimmunol. Neuroinflammation 2024; 11: e200235.
76. Morgan A, Li Y, Thompson NR, et al: Longitudinal Disability, Cognitive Impairment, and Mood Symptoms in Patients With Anti-NMDA Receptor Encephalitis. Neurology 2024; 102: e208019.

77. Griška V, Pranckevičienė A, Pakalnienė J, et al: Long-term neurological and neurocognitive impairments after tick-borne encephalitis in Lithuania - a prospective study. Infect. Dis. Lond. Engl. 2024; 56: 732–742.
78. Bergman K, Fowler Å, Ygberg S, et al: Neurocognitive outcome in children and adolescents following infectious encephalitis. Child Neuropsychol. J. Norm. Abnorm. Dev. Child. Adolesc. 2024; 30: 882–899.
79. Muñoz-Lopetegi A, Guasp M, Prades L, et al: Neurological, psychiatric, and sleep investigations after treatment of anti-leucine-rich glioma-inactivated protein 1 (LGI1) encephalitis in Spain: a prospective cohort study. Lancet Neurol. 2024; 23: 256–266.
80. Dwibedi B, Satapathy AK, Jain A, et al: Prevalence & clinical outcome of autoimmune encephalitis versus viral encephalitis in children with acute encephalitis syndrome: A prospective observational study. Indian J. Med. Res. 2024; 160: 217–225.
81. Mangioris G, Orozco E, Dubey D, et al: Long-Term Outcomes in Antibody-Negative Autoimmune Encephalitis: A Retrospective Study. Neurology 2024; 103: S83–S84.
82. Madani J, Yea C, Mahjoub A, et al: Clinical features and outcomes in children with seronegative autoimmune encephalitis. Dev. Med. Child Neurol. 2024; 66: 1310–1318.
83. Dumez P, Villagrán-García M, Bani-Sadr A, et al: Specific clinical and radiological characteristics of anti-NMDA receptor autoimmune encephalitis following herpes encephalitis. J. Neurol. 2024; 271: 6692–6701.
84. Benoit J, Muñiz-Castrillo S, Vogrig A, et al: Early-Stage Contactin-Associated Protein-like 2 Limbic Encephalitis: Clues for Diagnosis. Neurol. Neuroimmunol. Neuroinflammation 2023; 10: e200041.
85. Finke C, Kopp UA, Prüss H, et al: Cognitive deficits following anti-NMDA receptor encephalitis. J. Neurol. Neurosurg. Psychiatry 2012; 83: 195–198.
86. Heine J, Kopp UA, Klag J, et al: Long-Term Cognitive Outcome in Anti-N-Methyl-D-Aspartate Receptor Encephalitis. Ann. Neurol. 2021; 90: 949–961.
87. Byrne S, McCoy B, Lynch B, et al: Does early treatment improve outcomes in N-methyl-d-aspartate receptor encephalitis? Dev. Med. Child Neurol. 2014; 56: 794–796.
88. do Valle DA, Galeazzi JSP, Machado M de R, et al: Clinical variability of children with anti-N-methyl-D-aspartate receptor encephalitis in southern Brazil: a cases series and review of the literature. Neurol. Sci. Off. J. Ital. Neurol. Soc. Ital. Soc. Clin. Neurophysiol. 2019; 40: 351–356.
89. Raja P, Shamick B, Nitish LK, et al: Clinical characteristics, treatment and long-term prognosis in patients with anti-NMDAR encephalitis. Neurol. Sci. Off. J. Ital. Neurol. Soc. Ital. Soc. Clin. Neurophysiol. 2021; 42: 4683–4696.
90. Liu B, Liu J, Sun H, et al: Autoimmune encephalitis after Japanese encephalitis in children: A prospective study. J. Neurol. Sci. 2021; 424: 117394.
91. Wang Y, Zhang W, Yin J, et al: Anti-N-methyl-d-aspartate receptor encephalitis in children of Central South China: Clinical features, treatment, influencing factors, and outcomes. J. Neuroimmunol. 2017; 312: 59–65.
92. Yeshokumar A, Gordon-Lipkin E, Arenivas A, et al: Younger Age at Onset Is Associated With Worse Long-term Behavioral Outcomes in Anti-NMDA Receptor Encephalitis. Neurol. Neuroimmunol. Neuroinflammation 2022; 9: e200013.
93. Thakolwiboon S, Gilligan M, Orozco E, et al: Autoimmune encephalitis: recovery, residual symptoms and predictors of long-term sequelae. J. Neurol. Neurosurg. Psychiatry 2025. Available at: https://jnnp.bmj.com/content/early/2025/01/19/jnnp-2024-334957, accessed January 27, 2025.
94. Liu X, Zhang L, Chen C, et al: Long-term cognitive and neuropsychiatric outcomes in patients with anti-NMDAR encephalitis. Acta Neurol. Scand. 2019; 140: 414–421.
95. Galioto R, Aboseif A, Krishnan K, et al: Cognitive outcomes in anti-LGI-1 encephalitis. J. Int. Neuropsychol. Soc. JINS 2023; 29: 541–550.
96. Chen Z, Zhou J, Wu D, et al: Altered executive control network connectivity in anti-NMDA receptor encephalitis. Ann. Clin. Transl. Neurol. 2022; 9: 30–40.
97. Yeshokumar AK, Blum RA, Randell T, et al: Exploration of patient- and relative-reported outcomes of cognitive, emotional, and social function after encephalitis. Brain Inj. 2021; 35: 255–263.
98. Wang W, Li J-M, Hu F-Y, et al: Anti-NMDA receptor encephalitis: clinical characteristics, predictors of outcome and the knowledge gap in southwest China. Eur. J. Neurol. 2016; 23: 621–629.
99. Cai L, Liang Y, Huang H, et al: Cerebral functional activity and connectivity changes in anti-N-methyl-D-aspartate receptor encephalitis: A resting-state fMRI study. NeuroImage Clin. 2020; 25: 102189.
100. Meyding-Lamadé U, Jacobi C, Martinez-Torres F, et al: The German trial on Aciclovir and Corticosteroids in Herpes-simplex-virus-Encephalitis (GACHE): a multicenter, randomized, double-blind, placebo-controlled trial. Neurol. Res. Pract. 2019; 1: 26.
101. Argyropoulos GPD, Moore L, Loane C, et al: Pathologic tearfulness after limbic encephalitis. Neurology 2020; **94**: e1320–e1335.
